# MicroRNA-21 induces cisplatin resistance in head and neck squamous cell carcinoma

**DOI:** 10.1371/journal.pone.0267017

**Published:** 2022-04-14

**Authors:** Shuyan Sheng, Wenzhuo Su, Deshen Mao, Conghan Li, Xinyang Hu, Wanyu Deng, Yong Yao, Yongsheng Ji

**Affiliations:** 1 First Clinical Medical College, Anhui Medical University, Hefei, P. R China; 2 Second Clinical Medical College, Anhui Medical University, Hefei, P. R China; 3 College of Life Sciences, Anhui Medical University, Hefei, P. R China; 4 Division of Life Sciences and Medicine, University of Science and Technology of China, Hefei, Anhui, P.R China; Foshan University, CHINA

## Abstract

Drug resistance, either intrinsic or acquired, can impair treatment effects and result in increased cell motility and death. MicroRNA-21 (miR-21), a proto-oncogene, may facilitate the development or maintenance of drug resistance in cancer cells. Restoring drug sensitivity can improve therapeutic strategies, a possibility that requires functional evaluation and mechanistic exploration. For miR-21 detection, matched tissue samples from 30 head and neck squamous cell carcinoma (HNSCC) patients and 8 head and neck cancer (HNC) cell lines were obtained. Reverse transcription-PCR to detect expression, MTT and clonogenic assays to evaluate cell proliferation, apoptosis assays, resazurin cell viability assays, western blot and luciferase reporter assays to detect protein expression, and flow cytometry to analyse the cell cycle were adopted. Compared to the corresponding normal control (NC) tissues, 25 cancer tissues had miR-21 upregulation among the 30 matched pair tissues (25/30, 83.8%); furthermore, among the 8 HNC cell lines, miR-21 expression that was notably upregulated in three: UPCI-4B, UMSCC-1, and UPCI-15B. In both the UMSCC-1 and UPCI-4B cell lines, the miR-21 mimic enhanced cell proliferation with reduced apoptosis and increased viability, whereas the miR-21 inhibitor resulted in the opposite effects (all *P*<0.001); additionally, miR-21 directly targeted the tumour suppressor phosphatase and tensin homologue (PTEN) and inhibited PTEN expression. Furthermore, the miR-21 mimic induced cisplatin resistance, while the miR-21 inhibitor restored cisplatin sensitivity. Overexpression of miR-21 can enhance cell proliferation, reduce apoptosis, and induce drug resistance by inhibiting PTEN expression. Targeting miR-21 may facilitate cancer diagnosis, restore drug sensitivity, and improve therapeutic effects.

## Introduction

Head and neck cancer (HNC) ranks as the 6^th^ most common malignancy worldwide, with over 650,000 new cases diagnosed and 330, 000 deaths reported annually [1]. In the United States, there are approximately 53,000 new cases annually and 10,800 deaths from the disease, accounting for 3% of deaths from all malignancies [2]. Aggravating risk factors most frequently related to this disease include tobacco product (cigarettes, cigars, pipes) smoking, alcohol consumption, betel nut chewing, human papillomavirus (HPV) infection (especially for oropharyngeal cancers), and Epstein-Barr virus (EBV) infection (especially for nasopharyngeal cancers in Asia) [3]. Chronic exposure of the upper aerodigestive tract to these carcinogenic factors leads to dysplastic or premalignant lesions in the oropharyngeal mucosa and ultimately results in HNC. The relative prevalence of these risk factors contributes to the variations in the observed distribution of HNCs in different regions of the world.

Despite the decrease in the overall incidence of HNC in the United States over the past 30 years, there has been a drastic increase in the incidence of head and neck squamous cell carcinoma (HNSCC) of the base of the tongue and tonsils, especially in young to middle-aged populations due to the rising incidence of HPV-associated HNSCC [4]. Although HNC treatments include surgery, radiotherapy, chemotherapy, targeted therapy, or a combination of treatments, drug resistance results in a low survival rate in locoregionally advanced HNSCC [5]. However, the resistance mechanism remains unclear.

MicroRNAs (miRNAs), a class of small, single-stranded, ~19–23 nt RNA molecules, play pivotal roles in modulating neoplastic processes in cancers, including HNC [6] by regulating pathogenesis by inhibiting target genes [7]. The expression patterns of miRNAs may become robust biomarkers for the diagnosis and prognosis of HNC. In addition, miRNA therapy could be a novel strategy for HNC prevention and therapy [8]. Therefore, understanding how miRNAs are involved in HNC pathogenesis will help validate potential clinical applications to target these entities. Previous study identified that miR-21 was dramatically upregulated in HNC tissues compared with the corresponding matched NC tissues [9]. Due to the capability of increasing cell proliferation and invasion and reducing apoptosis by targeting the 3’-UTR of the genes tropomyosin 1 (TPM1), phosphatase and tensin homolog (PTEN), cyclin dependent kinase 2 associated protein 1 (CDK2AP1), reversion inducing cysteine rich protein with kazal motifs (RECK), and Clusterin (CLU) [9–12], upregulation of miR-21 has been associated with resistance to the favoring HNSCC, ovarian cancer, oral squamous cell cancer, gastric malignancy and non-small cell lung cancer (NSCLC) development and patients’ poor prognosis [13]. However, the function of miR-21 in in drug resistance in HNSCC tissues and cell lines remains unclear.

The purpose of this study was to address the functional importance and molecular mechanisms of miR-21 in regulating HNC cell growth and proliferation and the association of this miRNA with drug resistance.

## Materials and methods

### Patients and samples

Paired HNC tissue samples from thirty patients were obtained from the First Affiliated Hospital of Anhui Medical University with informed consent and agreement, and the characteristics of those patients had been described in the Table 1. All specimens were snap frozen in liquid nitrogen immediately after surgical resection and stored at -80°C until use. According to federal and institutional guidelines, written informed consent was obtained for each participant.

**Table 1:** Characteristics of the LC patients (n = 30).

Characteristic		n (%)
No. of patients		30
Age, y		
	Median	69
	Range	50–86
Sex		
	Male	24 (80)
	Female	6 (20)
Tobacco smoking		
	Ever	26 (87)
	Never	4 (13)
Primary tumor location		
	Tongue base	13 (43)
	Larynx	15 (50)
	Hypopharynx	2 (7)
TNM stage		
	T category	
	1–2	2 (7)
	3–4	28 (93)
	N category	
	0	10 (33)
	1–3	20 (67)
	M category	
	0	29 (97)
	1	1 (3)
Tumor stage		
	I-II	0 (0)
	III-IV	30 (100)
Histological differentiation		
	WD	12 (40)
	Moderately differentiated	14 (47)
	Poorly differentiated	4 (13)
HPV status		
	HPV positivity	2(7)
	HPV negativity	28(93)
Treatment		
	Surgery only	18 (60)
	Surgery + adjuvant treatment ^a^	12 (40)
	^a^radiotherapy and/or concurrent chemotherapy	

### Reagents and antibodies

The RT-PCR primer pairs for miR-21 (AM30102) and 5S rRNA (AM30302) were procured from Ambion Inc. (Austin, TX). The miRNA-21 mimics (MCH01533) were obtained from Applied Biological Materials Inc. (Richmond, BC, USA). The miRNA-21 inhibitors (MH10206) were purchased from Thermo Fisher Scientific (Waltham, MA, USA). The rabbit polyclonal anti-PTEN antibody (9552) was obtained from Cell Signaling Technology, Inc. (Danvers, MA, USA).

### Cell culture and transfection

The human HNC cell lines UMSCC-1, UMSCC-10A, UMSCC-22B, Cal33, UPCI-4B, UPCI-15B, 1483, and 686LN were authenticated and maintained as described previously [14]. Most of these cell lines were obtained from ATCC, and some were provided by Dr. Grandis (Department of Pharmacology and Chemical Biology, University of Pittsburgh School of Medicine, Pittsburgh, PA, USA). The in vitro experiments in cancer cell lines were approved by the medical ethics committees of Anhui Medical University and the University of Pittsburgh School of Medicine. UMSCC-1 is a unique human HNSCC cell line, and UPCI-4B (SCC090) is a cell line derived from squamous cell carcinoma of the base of the tongue. The miRNA-21 mimics, inhibitors, and negative controls were introduced into cells by transfection using LipofectamineTM 2000 (Invitrogen, Carlsbad, CA) according to the instructions of the manual as described previously [15]. Cells were incubated for 24 hours at 37°C in a humidified incubator containing 5% CO_2_ before testing and further experiments.

### Reverse transcription-PCR

As previous described [16], total RNA was extracted using a mirVana miRNA Isolation Kit (AM1560, Ambion Inc., Austin, TX). We used 15% denaturing polyacrylamide gel electrophoresis and spectrophotometry (Eppendorf BioPhotometer, Eppendorf, Hamburg, Germany) to assess the integrity of the extracted RNA. The absorbance at 260 nm (A260) was used to determine the RNA concentration, and the A260/A280 ratio was used to indicate the RNA purity. Moreover, the A260/A280 ratio was used to indicate the RNA purity. Reverse transcription-PCR (RT-PCR) was performed according to the instructions of the mirVana qRT-PCR miRNA Detection Kit (1558, Ambion, Austin, TX). The constructed primers of miR-21 and PTEN was shown in the Table 2.

**Table 2:** The constructed primers of miR-21 and PTEN for Q-RT-PCR.

Primer	forward	reverse
hu miR-21	5’-GCCAGGCATAGCTTATCAGACTG-3’	5’-CCACTGTCTAGCACGACACTAA-3’
hu PTEN	5’-AAAGGGACGAACTGGTGTAATG-3’	5’-TGGTCCTTACTTCCCCATAGAA-3’
hu β-actin	5’-GCATGGGTCAGAAGGATTCCT-3’	5’-TCGTCCCAGTTGGTGACGAT-3’
hu 5sRNA	5’-GTCTACGGCCATACCACCCTG-3’	5’-AAAGCCTACAGCACCCGGTAT-3’

### Cell proliferation assay

Cells transiently transfected with the miR-21 mimic, inhibitor, and negative controls were digested with trypsin and inoculated in 96-well plates at a concentration of 1×10^3^ cells/well after counting. Cell proliferation was monitored by an MTS assay using a CellTiter 96 AQueous Non-Radioactive Cell Proliferation Assay Kit (G5421, Promega, Madison, WI).

### Clonogenic assay

Cells transiently transfected with the miR-21 mimic, inhibitor, and negative controls were digested with trypsin and plated in 10-cm dishes at a concentration of 500 cells/well after counting. The experiments were performed as previously described [17].

### Flow cytometry assay

Flow cytometry assays were performed with BrdU and propidium iodide (PI) double staining (MP Biomedicals, Inc., Santa Ana, CA), as described previously [18]. Cisplatin was added at 5 ng/ml to 1×10^6^ cancer cells seeded in 10-cm dishes, and cells were harvested for flow cytometric detection after 24 hours of culture.

### Apoptosis detection assay

Cells transiently transfected with the miR-21 mimic, inhibitor, and negative controls were digested with trypsin and plated in 96-well plates at a concentration of 1×10^3^ cells/well after counting. A Cell Apoptosis PI Detection Kit (Cat. # L00311, Genscript, Piscataway, NJ) was used to evaluate apoptosis according to the instructions of the manual. Briefly, cells were incubated at 37°C in a humidified incubator containing 5% CO_2_. After incubation for the designated durations, cells were harvested by centrifugation at 2, 000 rpm for 5 minutes (min). Then, the cells were resuspended and adjusted to 1×10^6^ cells/ml after washing. Five microlitres of PI was added to 95 μl of the cell suspension prepared as described above, and the cells were incubated in the dark at room temperature for 5 min. After incubation, fluorescence was recorded using a H1 Synergy Hybrid Reader (BioTek, Winooski, VT). Experiments were carried out in quadruplicate, and the data were processed with GraphPad Prism. The results are shown as the mean±SD of three independent experiments. We adopted relative fluorescence units (RFUs) to express fluorescence values, as previously described [19].

### Western blotting

Cells transiently transfected with the miR-21 mimic, inhibitor, and negative controls were collected, and total cell lysates were prepared. After quantitation using a Bicinchoninic Acid Protein Assay Kit (Pierce, Rockford, IL), equal amounts of protein samples which was incubated with the primary antibody at 1: 2000 dilution in PBST, were subjected to western blotting for the measurement of PTEN expression levels.

### Luciferase reporter assay

pGL3-PTEN-3’-UTR vectors, which contained the putative binding site for miR-21 downstream of the stop codon in the pGL3 firefly luciferase reporter, were constructed. UMSCC-1 and UPCI-4B cells were plated at 1×10^6^ cells/well, and transfection was performed using one microgram of the pGL3-PTEN-3’-UTR vector and one microgram of the URL-TK Renilla luciferase expression vector (Promega, Beijing). Luciferase assays were performed 48 hours after transfection using a dual-luciferase reporter assay system (Promega, Beijing). Firefly luciferase activity was normalized to Renilla luciferase activity.

### Database selection and miRNA target prediction

During the process of identification, bioinformatic predictions were performed according to the mature miRNA sequence (5’-uagcuuaucagacugauguuga-3’) using TargetScan Release 7.2 (http://www.targetscan.org/vert_72/).

### Statistical analysis

Results are shown as the mean ± SD values. Student’s *t* test was used to evaluate comparisons unless another test is specified. (For all analyses, statistical significance was set at *P*<0.05, and all tests were two-sided. *, *P*< 0.05; **, *P*<0.01; ***, *P*<0.001; ****, *P*<0.0001; ns, nonsignificant.)

## Results

### Prevalence of miR-21 overexpression in HNSCC tissues and cell lines

The discovery of tumour-specific miRNA expression profiles with widespread dysregulation and differential expression of miRNA molecules can enhance the understanding of the diverse characteristics of cancers and their underlying mechanisms. In our previous study on miRNA expression profiles, based on matched cancer and adjacent NC tissues from five HNSCC patients, a total of 471 miRNA transcripts were identified (S1 Fig; S1 Table). Among these miRNAs, miR-21 was one of the most abundantly expressed miRNAs in tumour tissues compared to their NC counterparts. However, the underlying mechanisms of miR-21 remain unclear.

**Fig 1:**
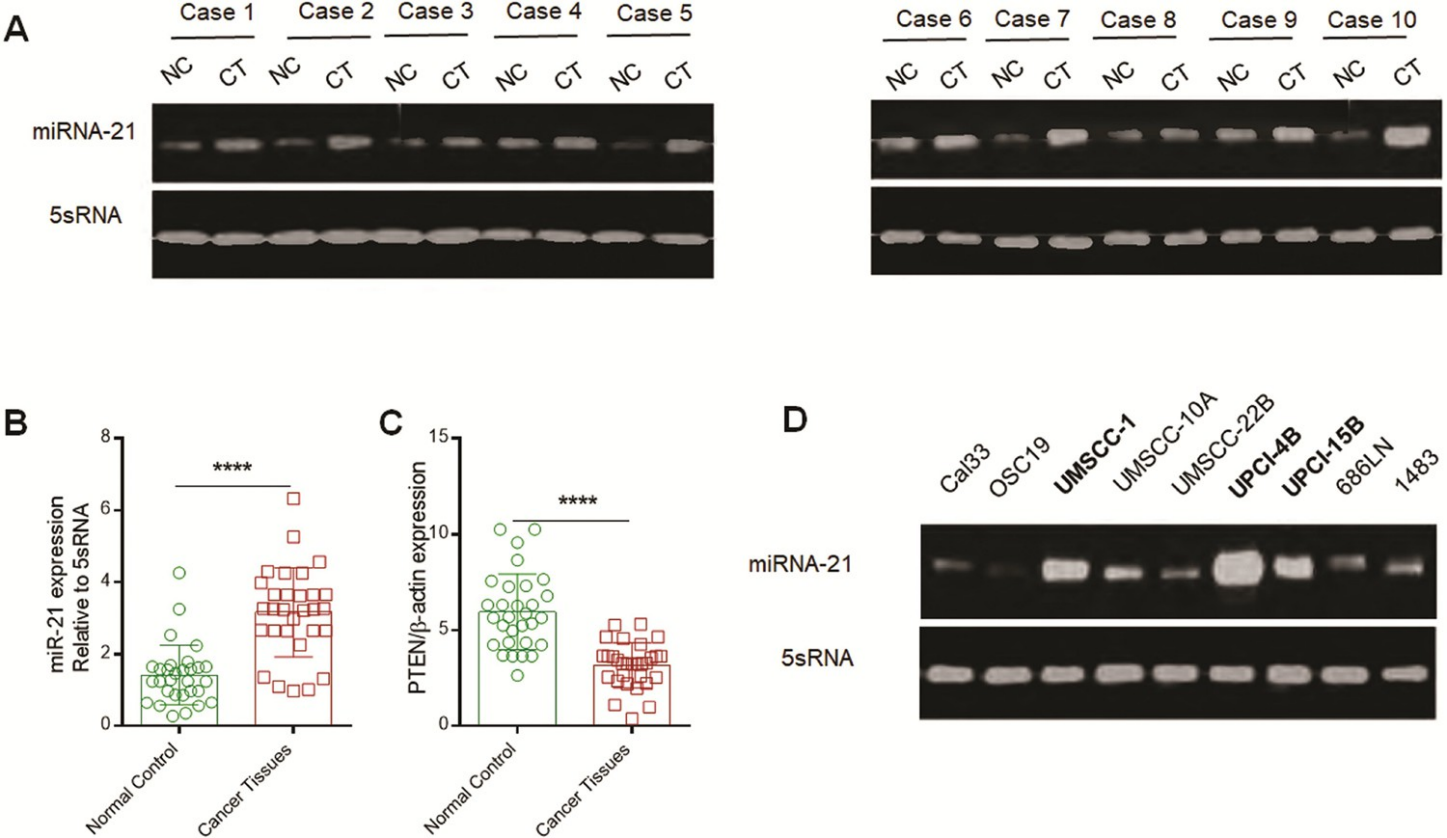
miR-21 overexpression is prevalent in human head and neck cancer. (A) RT-PCR analysis of miR-21 expression in matched head and neck tissue samples from patients, in which case-1, -2, and -3 and -6 were tongue base squamous cell cancer, case-4, -5, and -7 and -8 were laryngeal squamous cell cancer, and case-9 and -10 were hypopharyngeal squamous cell cancer. (B) and (C) Comparison of miR-21 and PTEN expression between cancer tissues and the corresponding matched adjacent NC tissues, respectively. *P*< 0.0001. (D) Among 8 head and neck SCC cell lines, miR-21 was significantly overexpressed in UMSCC-1, UPCI-4B, and UPCI-15B cells. CT, cancer tissue; NC, normal control, referring to the adjacent normal tissue. ****, *P*<0.0001.

In this study, we assessed the expression levels of miRNA-21 in matched tissues from 30 different individuals with HNSCC, among which 25 cancer tissues had miR-21 upregulation compared to the corresponding matched NC tissues (25/30, 83.3%) (Fig 1A and 1B). Furthermore, we detected miR-21 expression in 8 HNC cell lines and found that it was notably upregulated in UPCI-4B, UMSCC-1, and UPCI-15B cells (Fig 1C). These results demonstrated that miR-21 overexpression was prevalent among HNSCC tissues and HNSCC cell lines.

### MiR-21 enhances cell proliferation and reduces cell apoptosis

Since miR-21 upregulation in human head and neck SCC tissues and cell lines is a prevalent phenomenon, particularly in the cell lines UMSCC-1 and UPCI-4B, we both overexpressed and downregulated miR-21 and tested UMSCC-1 and UPCI-4B cell proliferation.

**Fig 2:**
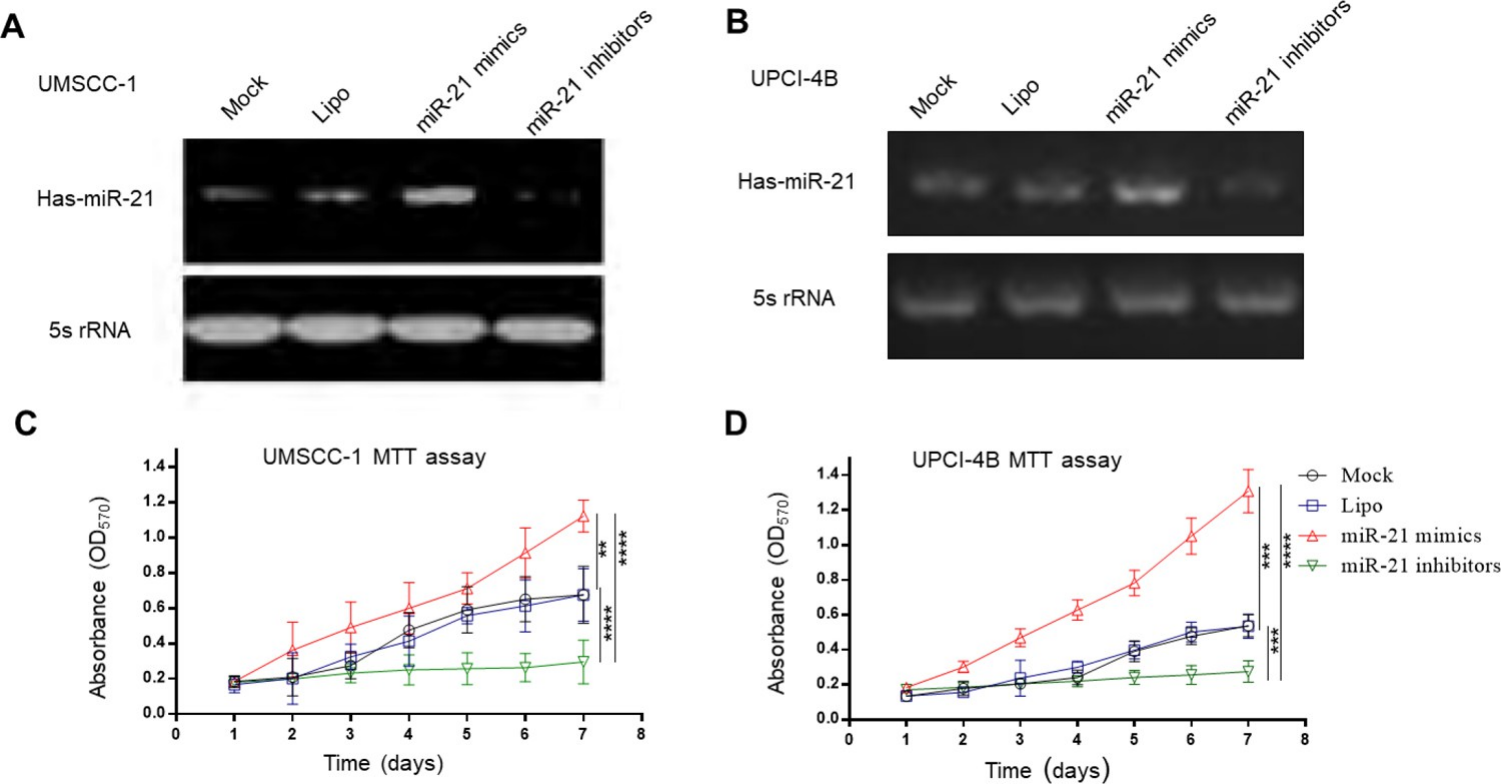
The MTT assay demonstrated that miR-21 can enhance cell growth and proliferation. (A) and (B) The miR-21 transfection efficiency was detected by PCR. UMSCC-1 and UPCI-4B cancer cells were transfected with the miR-21 mimic, inhibitor, and mock or liposome control. (C) and (D) MTT assays were performed to detect cell proliferation in UMSCC-1 and UPCI-4B cells, respectively. Compared to the corresponding mock or liposome control, the miR-21 mimic significantly increased cell proliferation, while the miR-21 inhibitor decreased cell growth in both cell lines. Mock, negative control; Lipo: Liposome control. **, *P*<0.01; ***, *P*<0.001; ****, *P*<0.0001. ns, nonsignificant.

Upon cell proliferation assessment, the MTT assay showed that the miR-21 mimic significantly enhanced cancer cell growth and proliferation, while the miR-21 inhibitor decreased cell growth and proliferation in both the UMSCC-1 and UPCI-4B cell lines (Fig 2C and 2D).

**Fig 3:**
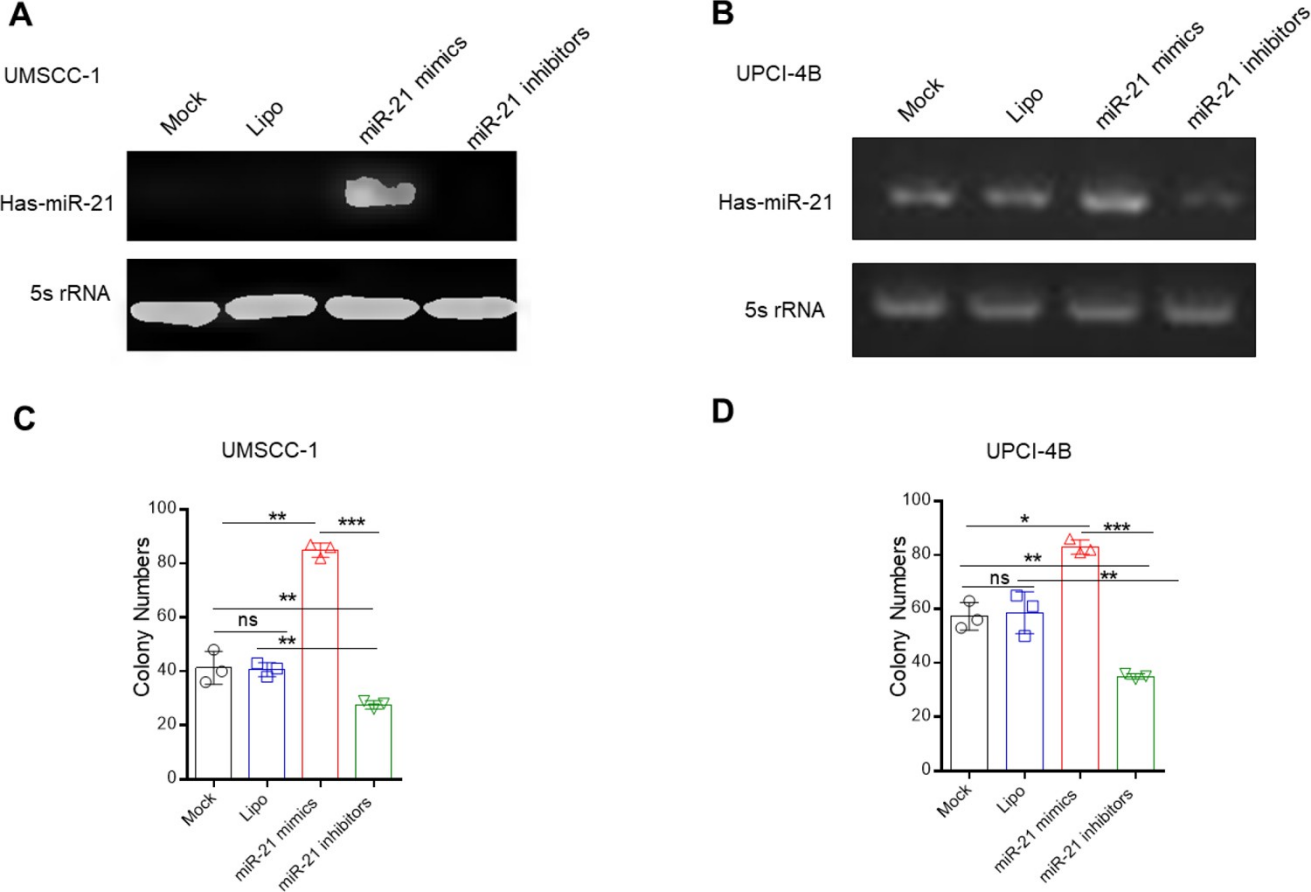
The clonogenic assay showed that miR-21 can enhance cell proliferation. (A) and (B) The miR-21 transfection efficiency was assessed by PCR in UMSCC-1 and UPCI-4B cells, respectively. (C) and (D) Clonogenic assays were performed to examine the effects of miR-21 in both cell lines. *, *P*< 0.05; **, *P*< 0.01; ***, *P*< 0.001. ns, nonsignificant.

Similarly, the clonogenic assay also demonstrated miR-21’s strong promotive effect on cell proliferation, while the miR-21 inhibitor exhibited different effects (Fig 3C and 3D).

**Fig 4:**
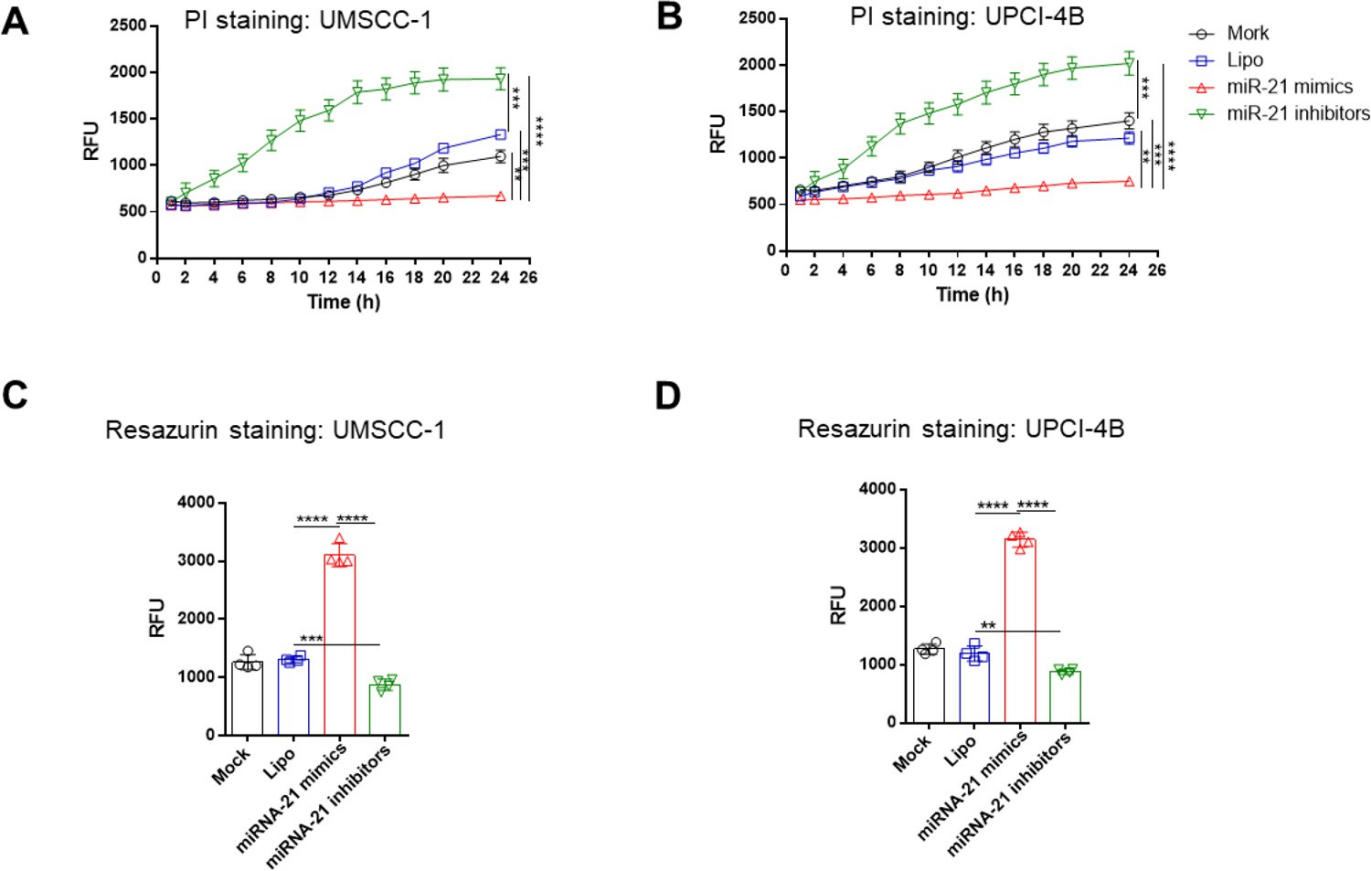
miR-21 can reduce apoptosis and enhance cell viability. (A) and (B) Apoptosis evaluation with Cell Apoptosis PI Detection assays showed that the miR-21 mimic significantly decreased apoptosis, while the miR-21 inhibitor increased cell apoptosis in both the UMSCC-1 and UPCI-4B cell lines, respectively. (C) and (D) Cell viability assessment with resazurin cell viability assays showed that the miR-21 mimic group had significantly more viable cells than the miR-21 inhibitor group in both the UMSCC-1 and UPCI-4B cell lines. **, *P*< 0.01; ***, *P*< 0.001; ****, *P*< 0.0001.

In the apoptosis detection assay, PI staining showed that the miR-21 mimic significantly reduced apoptosis, while the miR-21 inhibitor significantly increased apoptosis in both the UMSCC-1 and UPCI-4B cell lines (Fig 4A and 4B). Similarly, in the cell viability assay, the miR-21 inhibitor significantly decreased cell viability, while the miR-21 mimic increased cell viability in both cell lines (Fig 4C and 4D).

### miR-21 can promote cell cycle progression and induce cisplatin resistance

**Fig 5:**
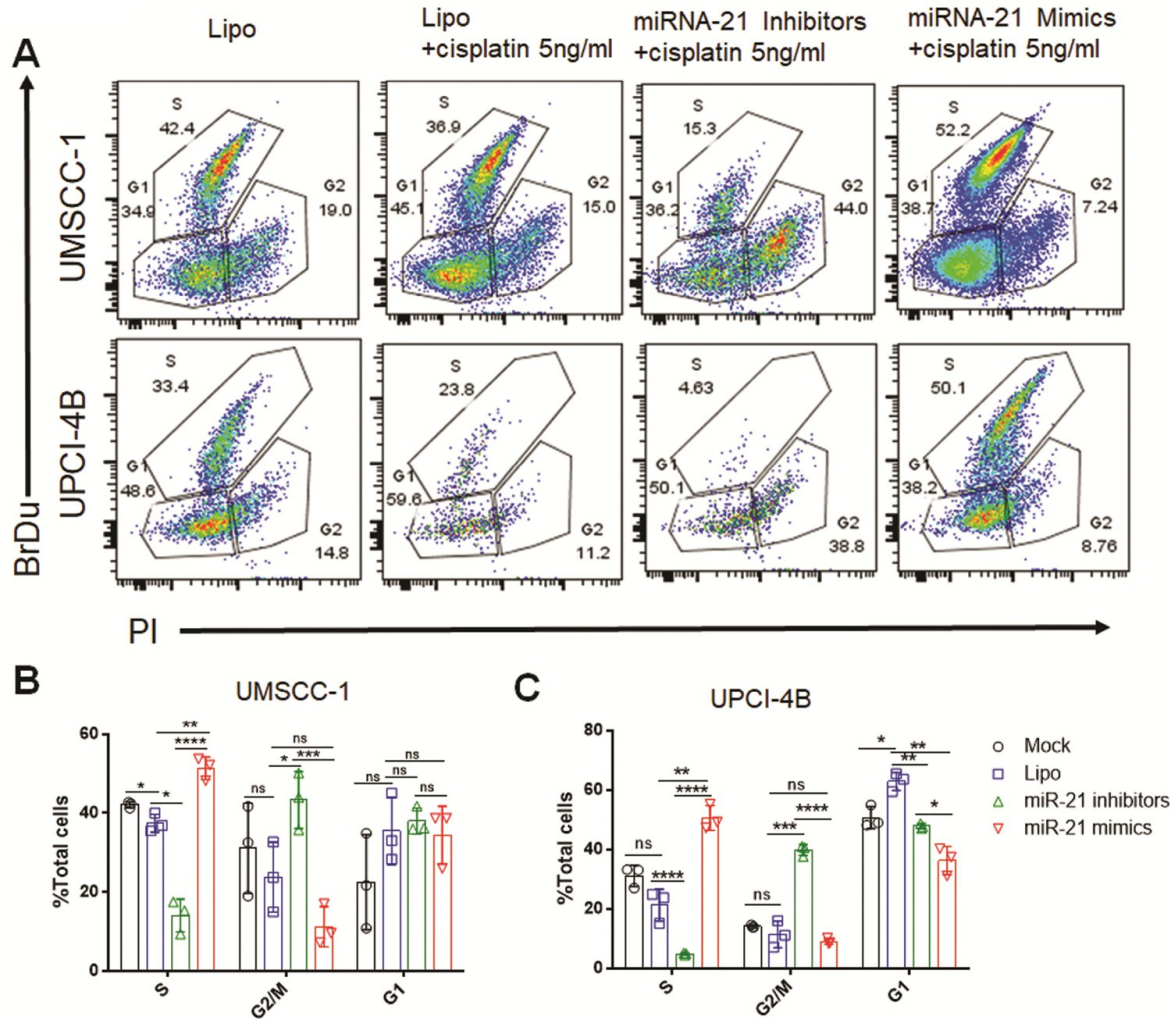
miR-21 can promote cell cycle progression and induce cisplatin resistance. (A) Cells were subjected to BrdU and PI double staining, and flow cytometry was used to analyse each phase of the cell cycle: cells in S phase (BrdU-positive), G2/M phases (BrdU-negative; 4N DNA content), and G1 phase (BrdU-negative; 2N DNA content). (B) and (C) In both UMSCC-1 and UPCI-4B cells treated with 5 ng/ml cisplatin during culture for 24 hours, miR-21 enhanced cell entry into S phase and induced cisplatin resistance, while the miR-21 inhibitor arrested cell cycle progression in G1, or G2, or M phase and restored cisplatin sensitivity. *, *P*< 0.05; **, *P*< 0.01; ***, *P*< 0.001; ****, *P*< 0.0001. ns, nonsignificant.

Since miR-21 had a strong promotive effect on cell growth and proliferation, we adopted a flow cytometry assay to test the impact of this miRNA on the cell cycle distribution via BrdU and PI co-staining. In both the UMSCC-1 and UPCI-4B cell lines, the miR-21 mimic enhanced cell entry into S (synthesis) phase and reduced cisplatin sensitivity, while the miR-21 inhibitor arrested cells in G2 and M phases and enhanced cisplatin sensitivity (Fig 5A, 5B and 5C, S2 and S3 Figs). This result implied that miR-21 can promote cell cycle progression to S phase and induce cisplatin resistance.

### miR-21 can directly target the 3’-UTR of PTEN and suppress PTEN expression

To investigate the underlying mechanism by which miR-21 enhances cell proliferation, reduces apoptosis, and induces cisplatin resistance, we adopted bioinformatic analysis to screen potential candidates. The screened candidates, as the targets of miR-21 and miR-21*, included a large number of oncogenic proteins and tumour suppressors, such as Ras, GRHL3, CHL1, PDCD4, PTEN, RECK and HNRPK (S2 Table).

**Fig 6:**
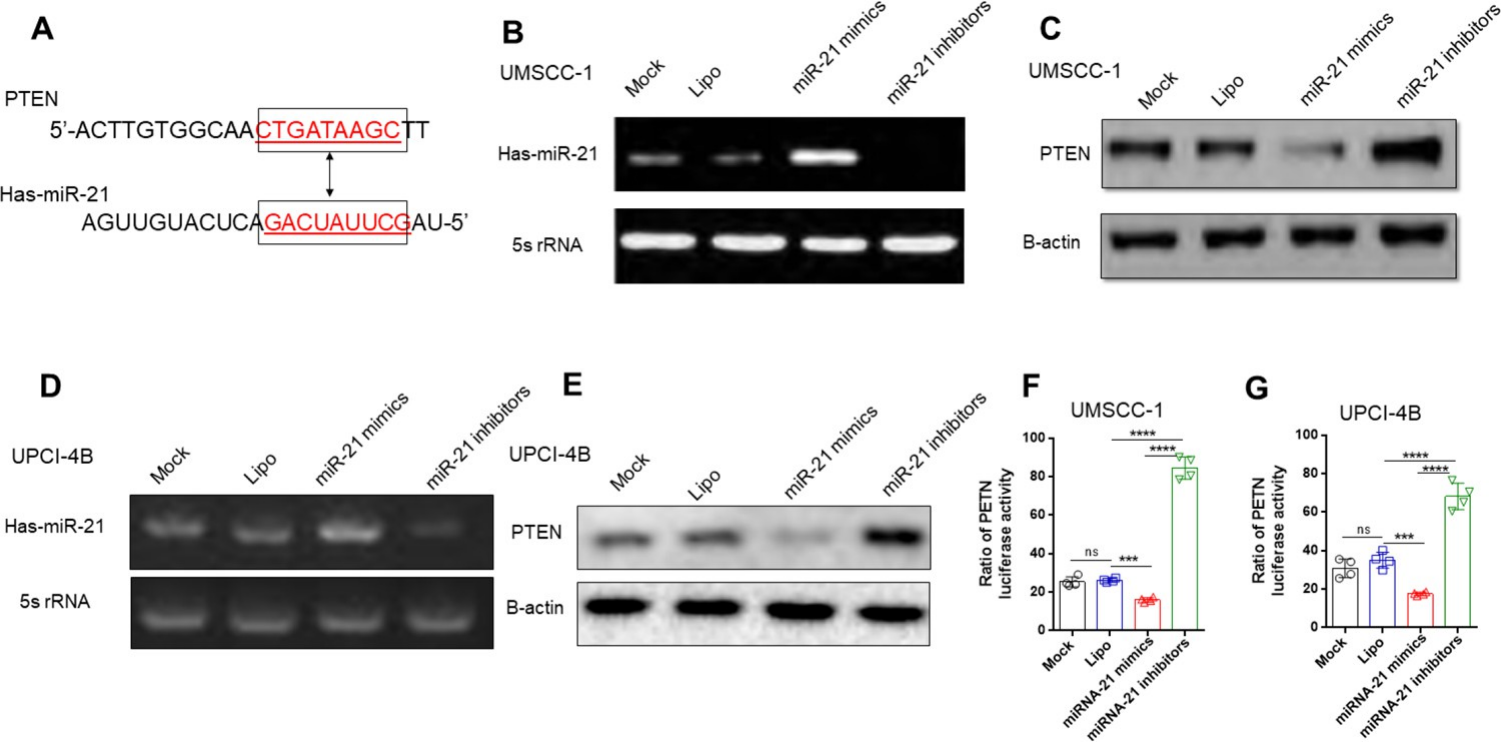
miR-21 can directly target the 3’-UTR of PTEN and suppress PTEN expression. (A) The binding structure of miR-21 and the 3’-UTR of PTEN. (B) RT-PCR analysis of miR-21 expression in UMSCC-1 cells transfected with the miR-21 mimic, inhibitor, or negative controls. (C) and (E) Western blot analysis of PTEN expression in UMSCC-1 and UPCI-4B cells, respectively, transfected with the miR-21 mimic, inhibitor, or negative controls showed that miR-21 significantly inhibited PTEN expression in both cell lines. (D) RT-PCR analysis of miR-21 expression in the UPCI-4B cell line. (F) and (G) UMSCC-1 and UPCI-4B cells were transfected with the miR-21 mimic, inhibitor, or negative controls; luciferase reporter assays showed that the miR-21 mimic significantly inhibited PTEN expression, while the inhibitor had the opposite effect. ***, *P*< 0.001; ****, *P*< 0.0001. ns, nonsignificant.

Structural analysis indicated that miR-21 can directly target the 3’-UTR of PTEN with the lowest free energy and that its binding to this site in PTEN may inhibit PTEN transcription (Fig 6A). We further assessed the protein level of PTEN with an immunoblot assay and found that the miR-21 mimic significantly inhibited PTEN expression, while the miR-21 inhibitor exerted the opposite effects in both cell lines (Fig 6C and 6E). In addition, the luciferase activity assay with the pGL3-PTEN-3’-UTR plasmid validated miR-21’s suppressive effect on PTEN expression (Fig 6F and 6G).

## Discussion

Numerous studies have demonstrated that miRNAs participate in tumorigenesis and that during this process, miRNAs play dual roles in either promoting or inhibiting tumour progression [20]. Based on sequence complementarity, these regulatory miRNAs act as guides to recognize specific mRNA sequences, resulting in site-specific cleavage or translational inhibition [21]. Among these miRNAs, miR-21 is a proto-oncogene, and its upregulation is often associated with ineffective treatment and unfavourable prognosis [22]. However, in HNC, the mechanisms of miR-21 remain unclear.

Cellular transcription driven by independent promotor elements can directly regulate miR-21 expression; however, members of the transforming growth factor β (TGF-β) family can also manipulate this regulation. Genome mapping and decoding indicate that pre-miR-21 is located on chromosome 17q23.2 [20]. Although pre-miR-21’s chromosomal location overlaps with that of TMEM49, pre-miR-21 has distinct promotor regions that contain specific binding sequences for transcriptional activators (activator protein 1, AP1) and suppressors (nuclear factor I, NFI). These binding structures indicate that miR-21 has independent promotor elements [23]. As an additional supplemental regulator, TGF-β can upregulate pre-miR-21 expression, while BMP6 (a member of the TGF-β family) can downregulate miR-21 expression [24]. These contrasting functions suggest that the members of the TGF-β family may establish an equilibrium state, which is disrupted within the tumour microenvironment because tumour cells can excessively express autocrine TGF-β1 [25, 26], which can lead to further production of an abundance of miR-21 and generate a feed-forward loop in cancer progression.

Upregulation of miR-21 can promote tumour progression because the majority of targets of miR-21 are tumour-suppressing genes. Studies of miR-21 upregulation and its tumour-promoting roles have been reported in various cancers, for example, in lung cancer [27], gastrointestinal cancer [28], colorectal cancer [29], breast cancer [30], glioblastoma [31], oral cancer, and HNSCC [32, 33]. Moreover, the target genes of miR-21 are generally PDCD4, RECK, p53 and PTEN, as well as their associated signalling pathways [33]. In the study of The Cancer Genome Atlas Network for HNSCC, the percentages (%) of PTEN genetic alterations in HPV (+) and HPV (-) tumours were 12% and 6%, respectively [34], and either PTEN mutation or inhibition could result in PIK3CA/CCND1/CDK6 pathway activation and thereby enhance cell proliferation [34]. In conclusion, these target genes and pathways perform tumour-suppressing functions, and their functional loss may result in tumour progression and distant metastasis. Consistent with previous studies, our results validated the upregulation of miR-21 and its tumour-promoting role in both the UMSCC-1 and UPCI-4B cell lines.

Inhibited PTEN expression resulted in the cisplatin resistance in HNSCC and recovery the PTEN function had high potential for better functional preservation and survival improvement. Previous studies had revealed that miR-21 could directly bind PTEN mRNA and thereby disrupt PTEN mRNA stability and inhibit PTEN protein expression. Specifically, PTEN is a lipid phosphatase that antagonizes phosphatidylinositol 3 kinase (PI3K) signaling by converting phosphatidylinositol trisphosphate (PIP3) to phosphatidylinositol bisphosphate (PIP2). This converting suppressed PIP3-dependent kinases (i.e., AKT, PDK1) that enhanced cell growth, protein synthesis, and cell cycling and migration and thereby inhibited tumor progression [35]. Importantly, cisplatin is widely used in cancer chemotherapy since its cellular toxicity function with DNA damage and inhibition of DNA synthesis, which further activates tyrosine kinase and AKT/PI3K signaling, while AKT/PI3K signaling can enhance cellular proliferation and cisplatin resistance [36]. Therefore, PTEN can inhibit AKT/PI3K signaling and enlarge the effects of cisplatin chemotherapy, while loss of PTEN resulted in chemotherapy resistance in various tumors [37]. Our finding had validated miR-21 can inhibit PTEN, and targeting miR-21 may have the potential to improve the effects of cisplatin chemotherapy.

MiR-21 overexpression can induce drug resistance, and this resistance mechanism, based primarily on miR-21’s target genes and pathways, has potential therapeutic relevance. In breast cancer, miR-21 can decrease PTEN and PDCD4 expression, whereas treatment with a miR-21 inhibitor combined with trastuzumab [38] or doxorubicin [39] can restore drug sensitivity. Similarly, in leukaemia, miR-21 upregulation leads to daunorubicin (DNR) resistance via PTEN suppression [40]. Moreover, miR-21 in glioblastoma cells can target LRRFIP1 and thereby facilitate NFκB pathway activation [41], while silencing miR-21 can strengthen the anti-tumour effects of sunitinib (a tyrosine kinase inhibitor) in U87 human glioblastoma cells [42]. Similar to these previous findings, our study on HNSCC cancer cell lines established that miR-21 overexpression can induce chemotherapeutic resistance to cisplatin via PTEN suppression, while miR-21 inhibition can restore drug sensitivity. In laryngeal cancer, concurrent chemo- and radiotherapy help to preserve laryngeal function [43], and restoration of drug sensitivity may contribute to reducing the motility of HNSCC cells.

Many studies have focused on tumour-associated miRNAs and their roles in carcinogenesis; however, quantitative studies of miR-21 inhibitors, from cellular mechanistic studies in vitro and tumour-bearing mouse models in vivo to clinical trials with full evaluation, have rarely been reported. Furthermore, miR-21 is essential for effective CD8+ T cell activation [44] and type 1 macrophage (M1) polarization and maintenance [45], which plays a crucial role in antitumour immunity. Similar to the side effects of chemotherapy on the immune system, miR-21 inhibitors may also inhibit these tumour-killing immune cells while suppressing tumour progression. Therefore, further observation of the effects of miR-21 inhibitors on tumour-infiltrating lymphocytes (TILs) in vitro and in vivo is suggested.

## Conclusion

In our studies, we confirmed that miR-21 overexpression is prevalent across HNSCC tissues and cell lines, and we further identified that miR-21 can enhance cell proliferation, reduce apoptosis and induce cisplatin resistance by inhibiting PTEN expression. Taken together, these findings indicate that miR-21 performs crucial functions in HNSCC cell proliferation and cisplatin resistance, while targeting miR-21 may facilitate the discovery of drugs with greater effectiveness and fewer side effects than current drugs, which may further improve the therapeutic strategies for HNSCC.

## Supporting information

S1 Fig Representative chip images demonstrate regions of miRNA transcripts. Five patient-matched snap-frozen head and neck tissue samples were employed for expression analysis of microRNAs by LC Sciences (http://www.lcsciences.com/; Houston, TX). Each chip included multiple control probes and 471 miRNA transcripts as listed in Sanger miRBase Release 9.2 (http://www.sanger.ac.uk/Software/Rfam/mirna/). T, tumor tissue; C, patient-matched adjacent normal control tissue. When Cy3 level was higher than Cy5 level, the color is green; when Cy3 level is equal to Cy5 level, the color is yellow; and when Cy5 level is higher than Cy3 level the color is red.
(TIF)

S2 Fig Flow cytometry analysis demonstrates that miR-21 can enhance cisplatin resistance (three independent experiments). (TIF)

S3 Fig The gating strategy divides cell cycling into three phases. (TIF)

S1 Table Differentially expressed miRNAs in five patient-matched samples. (DOCX)

S2 Table Predicted protein targets of miR-21 in human by bioinformatics analysis. (DOCX)

S1 Raw images (PDF)
