# Supplementary material for: MicroRNA-21 induces cisplatin resistance in head and neck squamous cell carcinoma
Source: PLoS One. 2022 Apr 14;17(4):e0267017. doi: 10.1371/journal.pone.0267017 (PMC9009694; doi:10.1371/journal.pone.0267017)
Supplement: S1 Table — (DOCX) [file pone.0267017.s004.docx]

**Table S1. Differentially expressed miRNAs in five patient-matched samples**

| **Chip 1_Sample A: HNSFT00587T-Cy3; Sample B: HNSFT00587C-Cy5** | | | | |
| --- | --- | --- | --- | --- |
| **No.** | **Probe_ID** | **Sample A Signal** | **Sample B Signal** | **log2 (B/A)** |
| 1 | hsa-miR-122a | 4,512.66 | 27.18 | -7.43 |
| 2 | hsa-miR-654 | 2,869.02 | 19.5 | -7.2 |
| 3 | hsa-miR-335 | 37.36 | 6,110.82 | 7.04 |
| 4 | hsa-miR-218 | 28.74 | 3,617.57 | 6.85 |
| 5 | hsa-miR-1 | 30.38 | 3,458.69 | 6.83 |
| 6 | hsa-miR-509 | 807.44 | 11.39 | -6.33 |
| 7 | hsa-miR-514 | 298.42 | 5.37 | -5.74 |
| 8 | hsa-miR-513 | 733.51 | 15.86 | -5.65 |
| 9 | hsa-miR-9 | 2,062.78 | 44.81 | -5.5 |
| 10 | hsa-miR-126* | 92.78 | 3,570.01 | 5.35 |
| 11 | hsa-miR-139 | 7.38 | 270.99 | 5.07 |
| 12 | hsa-miR-10a | 257.06 | 6,473.32 | 4.53 |
| 13 | hsa-miR-10b | 399.45 | 8,623.16 | 4.33 |
| 14 | hsa-miR-617 | 6.98 | 148.88 | 4.31 |
| 15 | hsa-miR-223 | 19,079.15 | 1,196.22 | -4.12 |
| 16 | hsa-miR-375 | 476.46 | 7,529.95 | 4 |
| 17 | hsa-miR-506 | 96.48 | 7.16 | -3.78 |
| 18 | hsa-miR-9* | 161.34 | 12.66 | -3.67 |
| 19 | hsa-miR-206 | 18.38 | 179.49 | 3.54 |
| 20 | hsa-miR-663 | 11,033.81 | 1,046.27 | -3.38 |
| 21 | hsa-miR-432 | 16.35 | 191.65 | 3.29 |
| 22 | hsa-miR-658 | 614.5 | 73.19 | -3.2 |
| 23 | hsa-miR-575 | 2,129.33 | 237.7 | -3.16 |
| 24 | hsa-miR-497 | 26.96 | 182.17 | 2.76 |
| 25 | hsa-miR-99a | 996.49 | 6,109.41 | 2.73 |
| 26 | hsa-miR-671 | 5,320.26 | 802.51 | -2.68 |
| 27 | hsa-miR-675 | 117.08 | 18.56 | -2.64 |
| 28 | hsa-miR-193b | 1,310.78 | 209.95 | -2.61 |
| 29 | hsa-miR-100 | 983.18 | 6,647.32 | 2.6 |
| 30 | hsa-miR-498 | 388.57 | 65.21 | -2.54 |
| 31 | hsa-miR-612 | 200.64 | 36.15 | -2.51 |
| 32 | hsa-miR-379 | 32.13 | 175.2 | 2.44 |
| 33 | hsa-miR-339 | 112.29 | 22.93 | -2.38 |
| 34 | hsa-miR-30e-3p | 126.77 | 629.31 | 2.37 |
| 35 | hsa-miR-199b | 97.25 | 490.77 | 2.37 |
| 36 | hsa-miR-768-5p | 689.73 | 3,401.51 | 2.3 |
| 37 | hsa-miR-98 | 3,069.37 | 15,621.79 | 2.3 |
| 38 | hsa-miR-25 | 15,492.16 | 3,226.57 | -2.29 |
| 39 | hsa-miR-93 | 4,274.28 | 863.61 | -2.28 |
| 40 | hsa-miR-30a-3p | 49.38 | 241.78 | 2.22 |
| 41 | hsa-miR-19a | 138.44 | 30.1 | -2.2 |
| 42 | hsa-miR-365 | 1,182.70 | 257.74 | -2.18 |
| 43 | hsa-miR-31 | 6,228.51 | 1,435.94 | -2.11 |
| 44 | hsa-miR-18a | 516.03 | 118.59 | -2.1 |
| 45 | hsa-miR-101 | 534.37 | 2,145.86 | 2.03 |
| 46 | hsa-miR-181d | 763.96 | 217.82 | -1.97 |
| 47 | hsa-miR-455 | 235.2 | 66.32 | -1.94 |
| 48 | hsa-miR-205 | 40,860.55 | 10,251.10 | -1.94 |
| 49 | hsa-miR-429 | 2,922.25 | 863.22 | -1.94 |
| 50 | hsa-miR-187 | 155.14 | 42.58 | -1.88 |
| 51 | hsa-miR-363 | 44.74 | 163.75 | 1.86 |
| 52 | hsa-miR-21 | 70,553.63 | 19,652.55 | -1.82 |
| 53 | hsa-miR-638 | 34,286.88 | 10,118.84 | -1.82 |
| 54 | hsa-miR-130b | 772.29 | 216.09 | -1.81 |
| 55 | hsa-miR-145 | 3,577.58 | 12,014.60 | 1.77 |
| 56 | hsa-miR-106b | 5,237.51 | 1,556.25 | -1.75 |
| 57 | hsa-miR-382 | 64.25 | 204.18 | 1.74 |
| 58 | hsa-miR-20b | 10,196.81 | 3,044.84 | -1.71 |
| 59 | hsa-miR-26b | 10,443.59 | 34,395.02 | 1.7 |
| 60 | hsa-miR-19b | 4,093.26 | 1,258.92 | -1.7 |
| 61 | hsa-miR-195 | 4,633.57 | 14,534.29 | 1.69 |
| 62 | hsa-miR-185 | 4,742.15 | 1,488.18 | -1.68 |
| 63 | hsa-miR-106a | 10,876.33 | 3,404.53 | -1.67 |
| 64 | hsa-miR-7 | 735.92 | 222.63 | -1.64 |
| 65 | hsa-miR-181b | 3,200.38 | 1,088.88 | -1.63 |
| 66 | hsa-miR-768-3p | 835.14 | 2,627.40 | 1.61 |
| 67 | hsa-miR-29b | 1,561.40 | 4,543.20 | 1.6 |
| 68 | hsa-miR-17-5p | 13,886.41 | 4,455.48 | -1.59 |
| 69 | hsa-miR-198 | 256.21 | 91.96 | -1.48 |
| 70 | hsa-miR-197 | 261.01 | 93.81 | -1.47 |
| 71 | hsa-miR-20a | 15,125.31 | 5,621.73 | -1.41 |
| 72 | hsa-miR-765 | 524.56 | 180.73 | -1.39 |
| 73 | hsa-miR-125b | 6,742.43 | 17,742.46 | 1.37 |
| 74 | hsa-miR-425-5p | 903.68 | 374.79 | -1.37 |
| 75 | hsa-miR-186 | 149.93 | 58.6 | -1.36 |
| 76 | hsa-miR-452 | 206.74 | 536.49 | 1.35 |
| 77 | hsa-miR-142-5p | 349.09 | 141.58 | -1.31 |
| 78 | hsa-miR-24 | 23,102.22 | 9,765.22 | -1.26 |
| 79 | hsa-miR-487b | 70.78 | 184.15 | 1.25 |
| 80 | hsa-miR-221 | 10,069.51 | 4,258.10 | -1.22 |
| 81 | hsa-miR-181a | 4,170.48 | 1,736.07 | -1.21 |
| 82 | hsa-miR-454-3p | 388.09 | 812.92 | 1.19 |
| 83 | hsa-miR-222 | 5,507.20 | 2,462.28 | -1.18 |
| 84 | hsa-miR-194 | 272.19 | 115.48 | -1.18 |
| 85 | hsa-let-7e | 11,204.43 | 27,763.12 | 1.16 |
| 86 | hsa-miR-422a | 100.46 | 219.58 | 1.16 |
| 87 | hsa-miR-203 | 19,976.46 | 43,969.50 | 1.15 |
| 88 | hsa-miR-155 | 2,196.34 | 4,673.80 | 1.15 |
| 89 | hsa-miR-148a | 4,442.86 | 9,305.44 | 1.13 |
| 90 | hsa-miR-130a | 570.66 | 1,192.91 | 1.12 |
| 91 | hsa-miR-320 | 11,021.67 | 4,995.70 | -1.09 |
| 92 | hsa-miR-34a | 378.21 | 778.23 | 1.09 |
| 93 | hsa-miR-125a | 3,452.26 | 7,205.37 | 1.06 |
| 94 | hsa-miR-103 | 7,121.37 | 3,591.61 | -1.01 |
| 95 | hsa-miR-92 | 11,518.12 | 5,488.95 | -1.01 |
| 96 | hsa-miR-92b | 6,939.66 | 3,535.64 | -1 |

| **Chip 2_Sample A: HNSFT00647T-Cy3; Sample B: HNSFT00647C-Cy5** | | | | |
| --- | --- | --- | --- | --- |
| **No.** | **Probe_ID** | **Sample A Signal** | **Sample B Signal** | **log2 (B/A)** |
| 1 | hsa-miR-1 | 4.88 | 577.67 | 6.81 |
| 2 | hsa-miR-375 | 9.14 | 356.57 | 5.23 |
| 3 | hsa-miR-21 | 40,117.68 | 2,568.90 | -3.97 |
| 4 | hsa-miR-34c | 104.36 | 6.99 | -3.9 |
| 5 | hsa-miR-186 | 156.79 | 15.2 | -3.37 |
| 6 | hsa-miR-181d | 681.12 | 66.01 | -3.19 |
| 7 | hsa-miR-429 | 1,843.26 | 215.14 | -3.18 |
| 8 | hsa-miR-374 | 1,031.41 | 112.61 | -3.17 |
| 9 | hsa-miR-150 | 323.92 | 2,164.03 | 2.74 |
| 10 | hsa-miR-654 | 170.31 | 25.49 | -2.68 |
| 11 | hsa-miR-25 | 5,296.50 | 911.72 | -2.5 |
| 12 | hsa-miR-34a | 189.53 | 1,042.32 | 2.48 |
| 13 | hsa-miR-10a | 260.57 | 1,351.06 | 2.48 |
| 14 | hsa-miR-223 | 2,606.22 | 450.33 | -2.45 |
| 15 | hsa-miR-200b | 17,924.78 | 3,285.12 | -2.44 |
| 16 | hsa-miR-30e-5p | 238.66 | 46.66 | -2.44 |
| 17 | hsa-miR-424 | 235.18 | 37.21 | -2.36 |
| 18 | hsa-miR-149 | 405.55 | 83.54 | -2.31 |
| 19 | hsa-miR-99a | 861.5 | 3,858.10 | 2.31 |
| 20 | hsa-miR-454-3p | 467.94 | 104.22 | -2.25 |
| 21 | hsa-miR-182 | 2,643.54 | 564.28 | -2.19 |
| 22 | hsa-miR-218 | 856.76 | 213.75 | -2.02 |
| 23 | hsa-miR-195 | 3,346.17 | 12,782.27 | 1.99 |
| 24 | hsa-miR-143 | 3,071.13 | 12,139.82 | 1.97 |
| 25 | hsa-miR-93 | 1,912.01 | 498.53 | -1.94 |
| 26 | hsa-miR-148b | 371.45 | 87.88 | -1.94 |
| 27 | hsa-miR-768-5p | 346.51 | 1,357.64 | 1.91 |
| 28 | hsa-miR-20b | 1,433.13 | 425.61 | -1.89 |
| 29 | hsa-miR-451 | 1,606.59 | 6,122.15 | 1.86 |
| 30 | hsa-miR-768-3p | 336.13 | 1,175.34 | 1.85 |
| 31 | hsa-miR-181b | 1,356.68 | 355.22 | -1.84 |
| 32 | hsa-miR-128b | 215.06 | 64.27 | -1.8 |
| 33 | hsa-miR-125a | 1,561.59 | 5,367.91 | 1.79 |
| 34 | hsa-miR-128a | 230.45 | 65.47 | -1.77 |
| 35 | hsa-miR-324-5p | 306.09 | 91.55 | -1.74 |
| 36 | hsa-miR-205 | 9,680.08 | 2,948.79 | -1.72 |
| 37 | hsa-miR-30b | 5,537.20 | 1,821.10 | -1.66 |
| 38 | hsa-miR-126 | 9,540.03 | 27,855.73 | 1.61 |
| 39 | hsa-miR-130b | 324.22 | 105.17 | -1.61 |
| 40 | hsa-miR-106b | 2,451.53 | 827.94 | -1.58 |
| 41 | hsa-miR-155 | 511.69 | 1,468.24 | 1.56 |
| 42 | hsa-miR-222 | 1,528.54 | 4,488.34 | 1.53 |
| 43 | hsa-miR-183 | 979.16 | 343.01 | -1.51 |
| 44 | hsa-miR-22 | 369.54 | 1,061.84 | 1.51 |
| 45 | hsa-miR-30c | 3,825.92 | 1,334.44 | -1.47 |
| 46 | hsa-miR-23a | 18,863.99 | 6,853.46 | -1.47 |
| 47 | hsa-miR-23b | 19,257.01 | 6,650.54 | -1.46 |
| 48 | hsa-miR-199a | 658.82 | 1,769.73 | 1.42 |
| 49 | hsa-miR-100 | 1,611.26 | 3,985.83 | 1.3 |
| 50 | hsa-miR-145 | 4,227.53 | 10,641.16 | 1.29 |
| 51 | hsa-miR-30a-5p | 3,220.15 | 1,345.49 | -1.25 |
| 52 | hsa-miR-200a | 4,710.79 | 2,058.17 | -1.19 |
| 53 | hsa-miR-200c | 12,908.77 | 5,719.63 | -1.16 |
| 54 | hsa-miR-342 | 1,386.85 | 677.54 | -1.03 |
| 55 | hsa-miR-27a | 8,435.77 | 4,087.26 | -1.02 |
| 56 | hsa-miR-152 | 1,489.63 | 2,937.14 | 1.01 |
| 57 | hsa-miR-28 | 1,026.98 | 500.52 | -1.01 |

| **Chip 3_Sample A: HNSFT00653T-Cy3; Sample B: HNSFT00653C-Cy5** | | | | |
| --- | --- | --- | --- | --- |
| **No.** | **Probe_ID** | **Sample A Signal** | **Sample B Signal** | **log2 (B/A)** |
| 1 | hsa-miR-9 | 985.62 | 6.63 | -7.22 |
| 2 | hsa-miR-126* | 94.76 | 4,008.85 | 5.49 |
| 3 | hsa-miR-9* | 60.13 | 1.72 | -5.09 |
| 4 | hsa-miR-617 | 7.17 | 143.67 | 4.44 |
| 5 | hsa-miR-139 | 18.64 | 425.76 | 4.42 |
| 6 | hsa-miR-375 | 286.86 | 5,899.93 | 4.35 |
| 7 | hsa-miR-1 | 58.15 | 1,299.80 | 4.35 |
| 8 | hsa-miR-218 | 51.67 | 913.67 | 4.26 |
| 9 | hsa-miR-654 | 248.32 | 12.54 | -4.17 |
| 10 | hsa-miR-650 | 628.17 | 44.32 | -3.9 |
| 11 | hsa-miR-7 | 1,691.31 | 136.2 | -3.61 |
| 12 | hsa-miR-10b | 764.38 | 6,985.07 | 3.31 |
| 13 | hsa-miR-10a | 404.71 | 2,858.95 | 2.79 |
| 14 | hsa-miR-34c | 87.46 | 13.53 | -2.68 |
| 15 | hsa-miR-193b | 2,111.92 | 326.89 | -2.67 |
| 16 | hsa-miR-224 | 669.83 | 3,979.11 | 2.57 |
| 17 | hsa-miR-206 | 29.52 | 157.58 | 2.46 |
| 18 | hsa-miR-31 | 5,939.88 | 1,189.94 | -2.33 |
| 19 | hsa-miR-452 | 87.51 | 453.73 | 2.32 |
| 20 | hsa-miR-95 | 42.36 | 199.33 | 2.25 |
| 21 | hsa-miR-374 | 286.93 | 1,358.84 | 2.19 |
| 22 | hsa-miR-584 | 36.56 | 172.78 | 2.11 |
| 23 | hsa-miR-149 | 1,463.29 | 348.45 | -2.07 |
| 24 | hsa-miR-365 | 845.31 | 204.12 | -2.05 |
| 25 | hsa-miR-98 | 1,862.48 | 6,571.86 | 1.97 |
| 26 | hsa-miR-199b | 139.22 | 496.03 | 1.93 |
| 27 | hsa-miR-30e-3p | 106.58 | 376.2 | 1.85 |
| 28 | hsa-miR-145 | 2,213.87 | 7,595.10 | 1.78 |
| 29 | hsa-miR-223 | 13,528.37 | 4,008.54 | -1.75 |
| 30 | hsa-miR-99a | 2,073.42 | 6,860.05 | 1.73 |
| 31 | hsa-miR-486 | 191.08 | 616.65 | 1.69 |
| 32 | hsa-miR-421 | 28.55 | 106.1 | 1.68 |
| 33 | hsa-miR-21 | 67,534.99 | 21,435.48 | -1.65 |
| 34 | hsa-miR-455 | 170.61 | 56.52 | -1.65 |
| 35 | hsa-miR-768-5p | 808.32 | 2,518.47 | 1.64 |
| 36 | hsa-miR-133b | 50.89 | 155.13 | 1.64 |
| 37 | hsa-miR-663 | 5,167.47 | 1,711.36 | -1.57 |
| 38 | hsa-miR-101 | 376.66 | 1,086.96 | 1.57 |
| 39 | hsa-miR-451 | 7,271.06 | 20,565.70 | 1.52 |
| 40 | hsa-miR-18a | 307.92 | 110.76 | -1.49 |
| 41 | hsa-miR-132 | 771.02 | 289.84 | -1.45 |
| 42 | hsa-miR-100 | 2,582.10 | 7,137.35 | 1.45 |
| 43 | hsa-miR-424 | 446.4 | 173.48 | -1.42 |
| 44 | hsa-miR-146a | 8,071.05 | 3,113.87 | -1.37 |
| 45 | hsa-miR-181d | 847.89 | 348.53 | -1.36 |
| 46 | hsa-miR-130b | 476.08 | 195.65 | -1.36 |
| 47 | hsa-miR-133a | 59.56 | 161.26 | 1.35 |
| 48 | hsa-miR-497 | 55.82 | 137.59 | 1.35 |
| 49 | hsa-miR-572 | 115.68 | 285.79 | 1.31 |
| 50 | hsa-miR-126 | 11,163.28 | 27,519.34 | 1.3 |
| 51 | hsa-miR-181b | 3,030.89 | 1,293.69 | -1.23 |
| 52 | hsa-miR-142-5p | 364.32 | 153.96 | -1.21 |
| 53 | hsa-miR-22 | 1,874.74 | 789.26 | -1.2 |
| 54 | hsa-miR-19b | 3,228.46 | 1,385.82 | -1.18 |
| 55 | hsa-miR-342 | 4,120.02 | 1,953.98 | -1.18 |
| 56 | hsa-miR-192 | 73.18 | 176.19 | 1.17 |
| 57 | hsa-miR-26b | 10,465.38 | 23,264.56 | 1.15 |
| 58 | hsa-miR-30c | 3,202.34 | 7,183.89 | 1.15 |
| 59 | hsa-miR-25 | 9,132.57 | 4,190.83 | -1.14 |
| 60 | hsa-miR-182 | 3,023.03 | 1,392.93 | -1.14 |
| 61 | hsa-miR-30a-5p | 1,996.78 | 4,396.63 | 1.14 |
| 62 | hsa-miR-146b | 7,824.40 | 3,864.87 | -1.07 |
| 63 | hsa-miR-29c | 2,474.50 | 5,228.38 | 1.06 |
| 64 | hsa-miR-335 | 317.5 | 573.09 | 1.06 |
| 65 | hsa-miR-181a | 4,057.30 | 1,883.81 | -1.05 |
| 66 | hsa-miR-183 | 768.47 | 375.9 | -1.02 |

| **Chip 4_Sample A: HNSFT00719T-Cy3; Sample B: HNSFT00719C-Cy5** | | | | |
| --- | --- | --- | --- | --- |
| **No.** | **Probe_ID** | **Sample A Signal** | **Sample B Signal** | **log2 (B/A)** |
| 1 | hsa-miR-1 | 258.39 | 31,317.54 | 7.07 |
| 2 | hsa-miR-133b | 54.81 | 5,730.46 | 6.62 |
| 3 | hsa-miR-133a | 46.74 | 3,821.33 | 6.3 |
| 4 | hsa-miR-206 | 175.42 | 10,658.74 | 5.89 |
| 5 | hsa-miR-7 | 541.58 | 12.6 | -5.43 |
| 6 | hsa-miR-499 | 32.17 | 1,045.33 | 4.97 |
| 7 | hsa-miR-31 | 3,183.26 | 124.12 | -4.64 |
| 8 | hsa-miR-155 | 9,693.42 | 477.62 | -4.34 |
| 9 | hsa-miR-424 | 1,532.09 | 117.46 | -3.72 |
| 10 | hsa-miR-146b | 8,385.61 | 808.04 | -3.38 |
| 11 | hsa-miR-95 | 53.05 | 501.65 | 3.35 |
| 12 | hsa-miR-422a | 26.32 | 223.01 | 3.27 |
| 13 | hsa-miR-378 | 9.8 | 78.43 | 3 |
| 14 | hsa-miR-450 | 78.56 | 11.53 | -2.98 |
| 15 | hsa-miR-572 | 31.53 | 213.18 | 2.79 |
| 16 | hsa-miR-368 | 86.29 | 599.01 | 2.77 |
| 17 | hsa-miR-126* | 475.66 | 3,157.70 | 2.76 |
| 18 | hsa-miR-637 | 11.27 | 74.72 | 2.75 |
| 19 | hsa-miR-139 | 28.35 | 171.23 | 2.69 |
| 20 | hsa-miR-422b | 198.5 | 1,254.95 | 2.64 |
| 21 | hsa-miR-375 | 109.3 | 626.28 | 2.47 |
| 22 | hsa-miR-503 | 100.62 | 16.24 | -2.46 |
| 23 | hsa-miR-342 | 5,483.67 | 963.77 | -2.46 |
| 24 | hsa-miR-21 | 73,021.53 | 12,934.91 | -2.43 |
| 25 | hsa-miR-150 | 6,222.11 | 1,202.73 | -2.38 |
| 26 | hsa-miR-203 | 6,427.90 | 30,752.57 | 2.25 |
| 27 | hsa-miR-376a | 38.73 | 175.75 | 2.23 |
| 28 | hsa-miR-663 | 454.13 | 2,147.81 | 2.19 |
| 29 | hsa-miR-142-5p | 105.86 | 24.87 | -2.18 |
| 30 | hsa-miR-602 | 20.48 | 88.45 | 2.11 |
| 31 | hsa-miR-149 | 46.22 | 169.54 | 2.07 |
| 32 | hsa-miR-29c | 1,274.50 | 5,412.70 | 2.06 |
| 33 | hsa-miR-186 | 20.96 | 83.03 | 2 |
| 34 | hsa-miR-146a | 7,546.21 | 1,903.94 | -1.96 |
| 35 | hsa-miR-15b | 8,520.47 | 2,199.88 | -1.9 |
| 36 | hsa-miR-382 | 319.44 | 90.17 | -1.82 |
| 37 | hsa-miR-299-5p | 76.16 | 244.31 | 1.79 |
| 38 | hsa-miR-638 | 5,276.30 | 18,131.00 | 1.78 |
| 39 | hsa-miR-132 | 650.11 | 197.56 | -1.71 |
| 40 | hsa-miR-455 | 92.7 | 31.08 | -1.69 |
| 41 | hsa-miR-30e-5p | 84.42 | 277.49 | 1.69 |
| 42 | hsa-miR-185 | 2,133.78 | 654.69 | -1.68 |
| 43 | hsa-miR-199b | 200.19 | 620.8 | 1.63 |
| 44 | hsa-miR-181d | 364.02 | 129.37 | -1.5 |
| 45 | hsa-miR-497 | 50.51 | 133.72 | 1.49 |
| 46 | hsa-miR-128b | 81.07 | 225.71 | 1.43 |
| 47 | hsa-miR-181b | 1,603.81 | 584.52 | -1.43 |
| 48 | hsa-miR-145 | 5,880.06 | 15,726.37 | 1.42 |
| 49 | hsa-miR-181a | 2,119.26 | 763.45 | -1.41 |
| 50 | hsa-miR-224 | 2,353.97 | 924.47 | -1.37 |
| 51 | hsa-miR-128a | 87.74 | 223.83 | 1.37 |
| 52 | hsa-miR-565 | 299.73 | 769.74 | 1.35 |
| 53 | hsa-miR-365 | 161.57 | 442.29 | 1.35 |
| 54 | hsa-miR-25 | 5,942.73 | 2,276.05 | -1.34 |
| 55 | hsa-miR-130b | 220.31 | 85.38 | -1.32 |
| 56 | hsa-miR-30a-5p | 1,952.08 | 4,974.19 | 1.31 |
| 57 | hsa-miR-454-3p | 515.28 | 210.32 | -1.28 |
| 58 | hsa-miR-182 | 1,070.39 | 463.01 | -1.23 |
| 59 | hsa-miR-214 | 13,758.63 | 6,027.67 | -1.18 |
| 60 | hsa-miR-671 | 427.95 | 930.48 | 1.18 |
| 61 | hsa-miR-15a | 5,494.33 | 2,446.22 | -1.14 |
| 62 | hsa-miR-486 | 1,284.20 | 606.93 | -1.06 |
| 63 | hsa-miR-99a | 2,174.11 | 4,408.02 | 1.04 |
| 64 | hsa-miR-106b | 1,483.26 | 770.74 | -1.02 |
| 65 | hsa-miR-29a | 7,053.63 | 14,200.83 | 1.01 |
| 66 | hsa-miR-575 | 125.17 | 236.84 | 1 |
| 67 | hsa-miR-126 | 11,869.36 | 23,589.75 | 1 |

| **Chip 5_Sample A: HNSFT00728T-Cy3; Sample B: HNSFT00728C-Cy5** | | | | |
| --- | --- | --- | --- | --- |
| **No.** | **Probe_ID** | **Sample A Signal** | **Sample B Signal** | **log2 (B/A)** |
| 1 | hsa-miR-122a | 898.49 | 6.98 | -7.03 |
| 2 | hsa-miR-139 | 11.22 | 313.75 | 4.85 |
| 3 | hsa-miR-133b | 140.72 | 3,821.19 | 4.73 |
| 4 | hsa-miR-9 | 60.69 | 3.37 | -4.48 |
| 5 | hsa-miR-133a | 130.98 | 2,723.19 | 4.45 |
| 6 | hsa-miR-7 | 109.68 | 5.24 | -4.39 |
| 7 | hsa-miR-29c | 546.64 | 8,236.52 | 3.98 |
| 8 | hsa-miR-126* | 61.29 | 796.16 | 3.82 |
| 9 | hsa-miR-499 | 8.56 | 109.33 | 3.73 |
| 10 | hsa-miR-375 | 128.59 | 1,620.45 | 3.69 |
| 11 | hsa-miR-1 | 2,421.02 | 24,670.59 | 3.39 |
| 12 | hsa-miR-450 | 114.04 | 12.74 | -3.34 |
| 13 | hsa-miR-182 | 5,294.58 | 561.44 | -3.29 |
| 14 | hsa-miR-21 | 82,130.68 | 8,099.10 | -3.28 |
| 15 | hsa-miR-10b | 335.14 | 2,854.85 | 3.16 |
| 16 | hsa-miR-183 | 1,643.01 | 191.11 | -3.1 |
| 17 | hsa-miR-101 | 198.48 | 1,593.65 | 3.01 |
| 18 | hsa-miR-765 | 224.85 | 30.7 | -2.93 |
| 19 | hsa-miR-486 | 72.34 | 547.13 | 2.93 |
| 20 | hsa-miR-150 | 349.26 | 2,446.91 | 2.85 |
| 21 | hsa-miR-565 | 82.28 | 490.8 | 2.63 |
| 22 | hsa-miR-155 | 3,155.78 | 517.84 | -2.55 |
| 23 | hsa-miR-451 | 1,152.45 | 6,384.27 | 2.52 |
| 24 | hsa-miR-422a | 36.34 | 216.89 | 2.52 |
| 25 | hsa-miR-149 | 2,646.74 | 489.55 | -2.41 |
| 26 | hsa-miR-424 | 1,105.90 | 236.65 | -2.41 |
| 27 | hsa-miR-146b | 3,571.22 | 705.04 | -2.35 |
| 28 | hsa-miR-30a-3p | 28.01 | 141.69 | 2.34 |
| 29 | hsa-miR-199b | 260.31 | 1,152.59 | 2.28 |
| 30 | hsa-miR-99a | 1,806.89 | 8,864.61 | 2.27 |
| 31 | hsa-miR-30e-5p | 75.66 | 349.61 | 2.23 |
| 32 | hsa-miR-30e-3p | 59.59 | 261.22 | 2.18 |
| 33 | hsa-miR-572 | 38.68 | 166.15 | 2.18 |
| 34 | hsa-miR-198 | 273.14 | 56.7 | -2.17 |
| 35 | hsa-miR-187 | 157.2 | 32.25 | -2.15 |
| 36 | hsa-miR-454-3p | 351.24 | 76.36 | -2.14 |
| 37 | hsa-miR-126 | 7,410.54 | 29,285.60 | 2.12 |
| 38 | hsa-miR-100 | 2,199.99 | 9,099.72 | 2.11 |
| 39 | hsa-miR-130b | 498.93 | 104.12 | -2.04 |
| 40 | hsa-miR-422b | 331.88 | 1,375.63 | 2.01 |
| 41 | hsa-miR-421 | 189.1 | 47.84 | -2 |
| 42 | hsa-miR-181d | 1,032.14 | 244.89 | -1.98 |
| 43 | hsa-miR-210 | 654.13 | 150 | -1.98 |
| 44 | hsa-miR-206 | 2,865.88 | 10,273.86 | 1.94 |
| 45 | hsa-miR-224 | 4,096.81 | 1,037.38 | -1.94 |
| 46 | hsa-miR-503 | 100.75 | 24.04 | -1.93 |
| 47 | hsa-miR-128b | 165.91 | 590.42 | 1.91 |
| 48 | hsa-miR-18a | 131.45 | 35.17 | -1.83 |
| 49 | hsa-miR-429 | 719.83 | 206.86 | -1.74 |
| 50 | hsa-miR-495 | 80.48 | 267.32 | 1.7 |
| 51 | hsa-miR-299-5p | 85.93 | 278.21 | 1.69 |
| 52 | hsa-miR-128a | 188.94 | 598.47 | 1.67 |
| 53 | hsa-miR-95 | 71.7 | 230.65 | 1.66 |
| 54 | hsa-miR-489 | 225.59 | 68.72 | -1.63 |
| 55 | hsa-miR-455 | 110.37 | 39.89 | -1.57 |
| 56 | hsa-miR-218 | 332.25 | 98.27 | -1.55 |
| 57 | hsa-miR-368 | 112.38 | 316.3 | 1.52 |
| 58 | hsa-miR-382 | 334.75 | 105.25 | -1.5 |
| 59 | hsa-miR-200b | 24,240.37 | 8,934.94 | -1.45 |
| 60 | hsa-miR-374 | 825.31 | 304 | -1.45 |
| 61 | hsa-miR-10a | 72.94 | 196.76 | 1.4 |
| 62 | hsa-miR-98 | 12,932.96 | 5,062.94 | -1.36 |
| 63 | hsa-miR-200c | 25,723.09 | 10,489.93 | -1.34 |
| 64 | hsa-miR-181b | 3,012.02 | 1,166.77 | -1.31 |
| 65 | hsa-miR-185 | 2,181.18 | 891.46 | -1.31 |
| 66 | hsa-miR-125b | 11,089.78 | 25,444.28 | 1.28 |
| 67 | hsa-miR-125a | 5,392.51 | 13,010.47 | 1.27 |
| 68 | hsa-let-7i | 29,292.16 | 11,324.76 | -1.27 |
| 69 | hsa-miR-200a | 1,677.29 | 654.81 | -1.27 |
| 70 | hsa-miR-148b | 797.88 | 341.06 | -1.22 |
| 71 | hsa-miR-660 | 95.04 | 219.59 | 1.19 |
| 72 | hsa-miR-29a | 9,459.48 | 21,223.40 | 1.17 |
| 73 | hsa-miR-28 | 2,157.66 | 889.46 | -1.17 |
| 74 | hsa-miR-452 | 259.44 | 110.14 | -1.15 |
| 75 | hsa-miR-29b | 399.61 | 877.3 | 1.12 |
| 76 | hsa-miR-25 | 7,249.77 | 3,206.25 | -1.12 |
| 77 | hsa-miR-181a | 2,961.16 | 1,319.05 | -1.12 |
| 78 | hsa-miR-151 | 1,187.64 | 541.63 | -1.1 |
| 79 | hsa-miR-30c | 4,020.57 | 8,238.73 | 1.09 |
| 80 | hsa-miR-93 | 1,661.61 | 819.99 | -1.08 |
| 81 | hsa-miR-152 | 5,651.42 | 2,623.03 | -1.06 |
| 82 | hsa-miR-335 | 723.64 | 337.29 | -1.03 |

* Mature miRNAs were sorted separately according to differential ratios. The ratio values were presented in log_2_ scale for quick and easy assessing differential direction as well as magnitude. A positive log_2_ value indicated an upper regulation while a negative log_2_ value indicated a down regulation.
