# Supplementary material for: MicroRNA-21 induces cisplatin resistance in head and neck squamous cell carcinoma
Source: PLoS One. 2022 Apr 14;17(4):e0267017. doi: 10.1371/journal.pone.0267017 (PMC9009694; doi:10.1371/journal.pone.0267017)
Supplement: S2 Table — (DOCX) [file pone.0267017.s005.docx]

**Table S2. Predicted protein targets of miR-21 in human by bioinformatics analysis**

| **Target gene** | **Representative transcript** | **Gene name** |
| --- | --- | --- |
| BRWD1 | ENST00000342449.3 | bromodomain and WD repeat domain containing 1 |
| ZNF367 | ENST00000375256.4 | zinc finger protein 367 |
| KRIT1 | ENST00000394507.1 | KRIT1, ankyrin repeat containing |
| IL12A | ENST00000466512.1 | interleukin 12A (natural killer cell stimulatory factor 1, cytotoxic lymphocyte maturation factor 1, p35) |
| FASLG | ENST00000340030.3 | Fas ligand (TNF superfamily, member 6) |
| FGF18 | ENST00000274625.5 | fibroblast growth factor 18 |
| CCL1 | ENST00000225842.3 | chemokine (C-C motif) ligand 1 |
| GPR64 | ENST00000379873.2 | G protein-coupled receptor 64 |
| AIM1L | ENST00000527815.1 | absent in melanoma 1-like |
| PLEKHA1 | ENST00000538022.1 | pleckstrin homology domain containing, family A (phosphoinositide binding specific) member 1 |
| RSAD2 | ENST00000382040.3 | radical S-adenosyl methionine domain containing 2 |
| YOD1 | ENST00000315927.4 | YOD1 deubiquitinase |
| PELI1 | ENST00000358912.4 | pellino E3 ubiquitin protein ligase 1 |
| TGFBI | ENST00000442011.2 | transforming growth factor, beta-induced, 68kDa |
| ARMCX1 | ENST00000372829.3 | armadillo repeat containing, X-linked 1 |
| MATN2 | ENST00000254898.5 | matrilin 2 |
| SKP2 | ENST00000274255.6 | S-phase kinase-associated protein 2, E3 ubiquitin protein ligase |
| NTF3 | ENST00000423158.3 | neurotrophin 3 |
| TIMP3 | ENST00000266085.6 | TIMP metallopeptidase inhibitor 3 |
| BEST3 | ENST00000488961.1 | bestrophin 3 |
| SMAD7 | ENST00000262158.2 | SMAD family member 7 |
| MSH2 | ENST00000233146.2 | mutS homolog 2 |
| RNFT1 | ENST00000442346.2 | ring finger protein, transmembrane 1 |
| SATB1 | ENST00000338745.6 | SATB homeobox 1 |
| PHF14 | ENST00000403050.3 | PHD finger protein 14 |
| FAM13A | ENST00000395002.2 | family with sequence similarity 13, member A |
| RP2 | ENST00000218340.3 | retinitis pigmentosa 2 (X-linked recessive) |
| RTN4 | ENST00000394609.2 | reticulon 4 |
| ARHGAP24 | ENST00000395184.1 | Rho GTPase activating protein 24 |
| UBE2D3 | ENST00000453744.2 | ubiquitin-conjugating enzyme E2D 3 |
| PPP1R3B | ENST00000310455.3 | protein phosphatase 1, regulatory subunit 3B |
| LRRC57 | ENST00000397130.3 | leucine rich repeat containing 57 |
| DUSP8 | ENST00000397374.3 | dual specificity phosphatase 8 |
| PDCD4 | ENST00000280154.7 | programmed cell death 4 (neoplastic transformation inhibitor) |
| SOX5 | ENST00000546136.1 | SRY (sex determining region Y)-box 5 |
| KBTBD7 | ENST00000379483.3 | kelch repeat and BTB (POZ) domain containing 7 |
| RMND5A | ENST00000283632.4 | required for meiotic nuclear division 5 homolog A (S. cerevisiae) |
| RAB22A | ENST00000244040.3 | RAB22A, member RAS oncogene family |
| PDZD8 | ENST00000334464.5 | PDZ domain containing 8 |
| OLR1 | ENST00000543993.1 | oxidized low density lipoprotein (lectin-like) receptor 1 |
| SLC2A4RG | ENST00000266077.2 | SLC2A4 regulator |
| SPRY1 | ENST00000394339.2 | sprouty homolog 1, antagonist of FGF signaling (Drosophila) |
| PFKM | ENST00000312352.7 | phosphofructokinase, muscle |
| S100A10 | ENST00000368811.3 | S100 calcium binding protein A10 |
| RALGPS2 | ENST00000367635.3 | Ral GEF with PH domain and SH3 binding motif 2 |
| GLIS2 | ENST00000262366.3 | GLIS family zinc finger 2 |
| KLF5 | ENST00000377687.4 | Kruppel-like factor 5 (intestinal) |
| SPRY2 | ENST00000377102.1 | sprouty homolog 2 (Drosophila) |
| ELF2 | ENST00000394235.2 | E74-like factor 2 (ets domain transcription factor) |
| RECK | ENST00000377966.3 | reversion-inducing-cysteine-rich protein with kazal motifs |
| PCBP1 | ENST00000303577.5 | poly(rC) binding protein 1 |
| SLC16A10 | ENST00000368850.3 | solute carrier family 16 (aromatic amino acid transporter), member 10 |
| ST3GAL6 | ENST00000265261.6 | ST3 beta-galactoside alpha-2,3-sialyltransferase 6 |
| KBTBD6 | ENST00000379485.1 | kelch repeat and BTB (POZ) domain containing 6 |
| PPP1R3A | ENST00000284601.3 | protein phosphatase 1, regulatory subunit 3A |
| SKI | ENST00000378536.4 | v-ski avian sarcoma viral oncogene homolog |
| PITX2 | ENST00000394595.3 | paired-like homeodomain 2 |
| DMRTC1B | ENST00000373532.3 | DMRT-like family C1B |
| STAG2 | ENST00000371160.1 | stromal antigen 2 |
| CD69 | ENST00000228434.3 | CD69 molecule |
| TADA2A | ENST00000394395.2 | transcriptional adaptor 2A |
| TRAPPC8 | ENST00000283351.4 | trafficking protein particle complex 8 |
| CPEB3 | ENST00000412050.4 | cytoplasmic polyadenylation element binding protein 3 |
| GMFB | ENST00000554908.1 | glia maturation factor, beta |
| PCSK6 | ENST00000348070.1 | proprotein convertase subtilisin/kexin type 6 |
| SESN1 | ENST00000436639.2 | sestrin 1 |
| PCBP2 | ENST00000455667.3 | poly(rC) binding protein 2 |
| HRK | ENST00000257572.5 | harakiri, BCL2 interacting protein (contains only BH3 domain) |
| BCL7A | ENST00000538010.1 | B-cell CLL/lymphoma 7A |
| GLCCI1 | ENST00000223145.5 | glucocorticoid induced transcript 1 |
| GABRB2 | ENST00000393959.1 | gamma-aminobutyric acid (GABA) A receptor, beta 2 |
| ARMC10 | ENST00000323716.3 | armadillo repeat containing 10 |
| LANCL1 | ENST00000443314.1 | LanC lantibiotic synthetase component C-like 1 (bacterial) |
| PAN3 | ENST00000282391.5 | PAN3 poly(A) specific ribonuclease subunit homolog (S. cerevisiae) |
| ST6GAL1 | ENST00000169298.3 | ST6 beta-galactosamide alpha-2,6-sialyltranferase 1 |
| ASF1A | ENST00000229595.5 | anti-silencing function 1A histone chaperone |
| PBRM1 | ENST00000356770.4 | polybromo 1 |
| JHDM1D | ENST00000397560.2 | jumonji C domain containing histone demethylase 1 homolog D (S. cerevisiae) |
| JAG1 | ENST00000254958.5 | jagged 1 |
| MAP2K3 | ENST00000342679.4 | mitogen-activated protein kinase kinase 3 |
| GRAMD3 | ENST00000285689.3 | GRAM domain containing 3 |
| CHIC1 | ENST00000373504.6 | cysteine-rich hydrophobic domain 1 |
| TMEM170A | ENST00000357613.4 | transmembrane protein 170A |
| DCAF7 | ENST00000310827.4 | DDB1 and CUL4 associated factor 7 |
| HIPK3 | ENST00000303296.4 | homeodomain interacting protein kinase 3 |
| TIAM1 | ENST00000286827.3 | T-cell lymphoma invasion and metastasis 1 |
| OSR1 | ENST00000272223.2 | odd-skipped related 1 (Drosophila) |
| MAP3K1 | ENST00000399503.3 | mitogen-activated protein kinase kinase kinase 1, E3 ubiquitin protein ligase |
| C10orf12 | ENST00000286067.2 | chromosome 10 open reading frame 12 |
| CASKIN1 | ENST00000343516.6 | CASK interacting protein 1 |
| MBLAC2 | ENST00000316610.6 | metallo-beta-lactamase domain containing 2 |
| RBPJ | ENST00000504907.1 | recombination signal binding protein for immunoglobulin kappa J region |
| DAZL | ENST00000250863.8 | deleted in azoospermia-like |
| NFIB | ENST00000397575.3 | nuclear factor I/B |
| XKR6 | ENST00000304437.2 | XK, Kell blood group complex subunit-related family, member 6 |
| ERG | ENST00000398905.1 | v-ets avian erythroblastosis virus E26 oncogene homolog |
| ALX1 | ENST00000316824.3 | ALX homeobox 1 |
| PLAG1 | ENST00000316981.3 | pleiomorphic adenoma gene 1 |
| CNTFR | ENST00000351266.4 | ciliary neurotrophic factor receptor |
| RP11-766F14.2 | ENST00000511828.1 | Protein LOC285556 |
| PSRC1 | ENST00000409267.1 | proline/serine-rich coiled-coil 1 |
| ZFP36L2 | ENST00000282388.3 | ZFP36 ring finger protein-like 2 |
| ACVR1C | ENST00000243349.8 | activin A receptor, type IC |
| THRB | ENST00000396671.2 | thyroid hormone receptor, beta |
| FBXO11 | ENST00000402508.1 | F-box protein 11 |
| SRL | ENST00000399609.3 | sarcalumenin |
| XKR8 | ENST00000373884.5 | XK, Kell blood group complex subunit-related family, member 8 |
| CSRNP3 | ENST00000314499.7 | cysteine-serine-rich nuclear protein 3 |
| FAM46A | ENST00000369754.3 | family with sequence similarity 46, member A |
| ZBTB47 | ENST00000457842.3 | zinc finger and BTB domain containing 47 |
| NAA50 | ENST00000240922.3 | N(alpha)-acetyltransferase 50, NatE catalytic subunit |
| LEMD3 | ENST00000308330.2 | LEM domain containing 3 |
| STAT3 | ENST00000585517.1 | signal transducer and activator of transcription 3 (acute-phase response factor) |
| ZSWIM6 | ENST00000252744.5 | zinc finger, SWIM-type containing 6 |
| SESTD1 | ENST00000428443.3 | SEC14 and spectrin domains 1 |
| RBM22 | ENST00000199814.4 | RNA binding motif protein 22 |
| RASA2 | ENST00000286364.3 | RAS p21 protein activator 2 |
| PAG1 | ENST00000220597.4 | phosphoprotein associated with glycosphingolipid microdomains 1 |
| SLC8A3 | ENST00000356921.2 | solute carrier family 8 (sodium/calcium exchanger), member 3 |
| KLHL42 | ENST00000381271.2 | kelch-like family member 42 |
| PURA | ENST00000331327.3 | purine-rich element binding protein A |
| ADNP | ENST00000371602.4 | activity-dependent neuroprotector homeobox |
| SRSF3 | ENST00000373715.6 | serine/arginine-rich splicing factor 3 |
| AP1AR | ENST00000274000.5 | adaptor-related protein complex 1 associated regulatory protein |
| NFIA | ENST00000403491.3 | nuclear factor I/A |
| CCL22 | ENST00000219235.4 | chemokine (C-C motif) ligand 22 |
| VTA1 | ENST00000367630.4 | vesicle (multivesicular body) trafficking 1 |
| ARHGEF12 | ENST00000397843.2 | Rho guanine nucleotide exchange factor (GEF) 12 |
| PIK3R1 | ENST00000521381.1 | phosphoinositide-3-kinase, regulatory subunit 1 (alpha) |
| WWP1 | ENST00000517970.1 | WW domain containing E3 ubiquitin protein ligase 1 |
| PRRG4 | ENST00000257836.3 | proline rich Gla (G-carboxyglutamic acid) 4 (transmembrane) |
| ZNF704 | ENST00000327835.3 | zinc finger protein 704 |
| MAPK10 | ENST00000395169.3 | mitogen-activated protein kinase 10 |
| SOCS6 | ENST00000397942.3 | suppressor of cytokine signaling 6 |
| FRS2 | ENST00000550389.1 | fibroblast growth factor receptor substrate 2 |
| VASH2 | ENST00000366968.4 | vasohibin 2 |
| NELL2 | ENST00000395487.2 | NEL-like 2 (chicken) |
| NIPAL1 | ENST00000295461.5 | NIPA-like domain containing 1 |
| SOX2 | ENST00000325404.1 | SRY (sex determining region Y)-box 2 |
| ZBTB8A | ENST00000316459.4 | zinc finger and BTB domain containing 8A |
| PVRL3 | ENST00000319792.3 | poliovirus receptor-related 3 |
| EIF4EBP2 | ENST00000373218.4 | eukaryotic translation initiation factor 4E binding protein 2 |
| CNOT8 | ENST00000285896.6 | CCR4-NOT transcription complex, subunit 8 |
| LIFR | ENST00000263409.4 | leukemia inhibitory factor receptor alpha |
| KLF6 | ENST00000542957.1 | Kruppel-like factor 6 |
| RAD21 | ENST00000297338.2 | RAD21 homolog (S. pombe) |
| CADM2 | ENST00000383699.3 | cell adhesion molecule 2 |
| GATAD2B | ENST00000368655.4 | GATA zinc finger domain containing 2B |
| PI15 | ENST00000260113.2 | peptidase inhibitor 15 |
| FGD4 | ENST00000427716.2 | FYVE, RhoGEF and PH domain containing 4 |
| MSX1 | ENST00000382723.4 | msh homeobox 1 |
| SMARCD1 | ENST00000394963.4 | SWI/SNF related, matrix associated, actin dependent regulator of chromatin, subfamily d, member 1 |
| MRPL49 | ENST00000279242.2 | mitochondrial ribosomal protein L49 |
| CCR7 | ENST00000246657.2 | chemokine (C-C motif) receptor 7 |
| SLC10A7 | ENST00000264986.3 | solute carrier family 10, member 7 |
| MIA3 | ENST00000344922.5 | melanoma inhibitory activity family, member 3 |
| NPAS3 | ENST00000346562.2 | neuronal PAS domain protein 3 |
| MARCH5 | ENST00000358935.2 | membrane-associated ring finger (C3HC4) 5 |
| NUDT5 | ENST00000491614.1 | nudix (nucleoside diphosphate linked moiety X)-type motif 5 |
| ZCCHC3 | ENST00000382352.3 | zinc finger, CCHC domain containing 3 |
| PPP3CA | ENST00000512215.1 | protein phosphatase 3, catalytic subunit, alpha isozyme |
| SPG20 | ENST00000438666.2 | spastic paraplegia 20 (Troyer syndrome) |
| MXD1 | ENST00000264444.2 | MAX dimerization protein 1 |
| VHL | ENST00000256474.2 | von Hippel-Lindau tumor suppressor, E3 ubiquitin protein ligase |
| RPRD1A | ENST00000399022.4 | regulation of nuclear pre-mRNA domain containing 1A |
| CBX4 | ENST00000269397.4 | chromobox homolog 4 |
| CHD7 | ENST00000423902.2 | chromodomain helicase DNA binding protein 7 |
| BOLL | ENST00000392296.4 | bol, boule-like (Drosophila) |
| RPP25 | ENST00000322177.5 | ribonuclease P/MRP 25kDa subunit |
| ABHD10 | ENST00000494817.1 | abhydrolase domain containing 10 |
| IL6ST | ENST00000381287.4 | interleukin 6 signal transducer (gp130, oncostatin M receptor) |
| SERP1 | ENST00000239944.2 | stress-associated endoplasmic reticulum protein 1 |
| RYBP | ENST00000477973.2 | RING1 and YY1 binding protein |
| TAGAP | ENST00000367066.3 | T-cell activation RhoGTPase activating protein |
| RASEF | ENST00000376447.3 | RAS and EF-hand domain containing |
| JPH1 | ENST00000342232.4 | junctophilin 1 |
| FBXO28 | ENST00000424254.2 | F-box protein 28 |
| MBNL1 | ENST00000357472.3 | muscleblind-like splicing regulator 1 |
| RAD51L3-RFFL | ENST00000593039.1 | Uncharacterized protein |
| GTPBP1 | ENST00000216044.5 | GTP binding protein 1 |
| MICALL1 | ENST00000215957.6 | MICAL-like 1 |
| EPHA4 | ENST00000281821.2 | EPH receptor A4 |
| UBR3 | ENST00000272793.5 | ubiquitin protein ligase E3 component n-recognin 3 (putative) |
| NEGR1 | ENST00000357731.5 | neuronal growth regulator 1 |
| VCL | ENST00000372755.3 | vinculin |
| FAM63B | ENST00000559228.1 | family with sequence similarity 63, member B |
| KLHL15 | ENST00000328046.8 | kelch-like family member 15 |
| ESM1 | ENST00000381405.4 | endothelial cell-specific molecule 1 |
| POC1B | ENST00000378528.2 | POC1 centriolar protein B |
| BTG2 | ENST00000290551.4 | BTG family, member 2 |
| LRP6 | ENST00000261349.4 | low density lipoprotein receptor-related protein 6 |
| TESK2 | ENST00000372084.1 | testis-specific kinase 2 |
| COL4A1 | ENST00000375820.4 | collagen, type IV, alpha 1 |
| RAVER2 | ENST00000371072.4 | ribonucleoprotein, PTB-binding 2 |
| FGF7 | ENST00000267843.4 | fibroblast growth factor 7 |
| MICALCL | ENST00000256186.2 | MICAL C-terminal like |
| RFFL | ENST00000315249.7 | ring finger and FYVE-like domain containing E3 ubiquitin protein ligase |
| CDC25A | ENST00000302506.3 | cell division cycle 25A |
| MBNL3 | ENST00000370839.3 | muscleblind-like splicing regulator 3 |
| RGS7BP | ENST00000334025.2 | regulator of G-protein signaling 7 binding protein |
| KPNA4 | ENST00000334256.4 | karyopherin alpha 4 (importin alpha 3) |
| AKIRIN1 | ENST00000432648.3 | akirin 1 |
| ROR1 | ENST00000371079.1 | receptor tyrosine kinase-like orphan receptor 1 |
| CCL20 | ENST00000409189.3 | chemokine (C-C motif) ligand 20 |
| PURG | ENST00000475541.1 | purine-rich element binding protein G |
| PIKFYVE | ENST00000264380.4 | phosphoinositide kinase, FYVE finger containing |
| ANKRD46 | ENST00000335659.3 | ankyrin repeat domain 46 |
| AIF1L | ENST00000372300.1 | allograft inflammatory factor 1-like |
| TET1 | ENST00000373644.4 | tet methylcytosine dioxygenase 1 |
| HNRNPK | ENST00000376281.4 | heterogeneous nuclear ribonucleoprotein K |
| SOWAHC | ENST00000356454.3 | sosondowah ankyrin repeat domain family member C |
| EDNRB | ENST00000377211.4 | endothelin receptor type B |
| SLMAP | ENST00000295951.3 | sarcolemma associated protein |
| C11orf87 | ENST00000327419.6 | chromosome 11 open reading frame 87 |
| MKX | ENST00000375790.5 | mohawk homeobox |
| GLYR1 | ENST00000436648.5 | glyoxylate reductase 1 homolog (Arabidopsis) |
| PTPN14 | ENST00000366956.5 | protein tyrosine phosphatase, non-receptor type 14 |
| PDZD2 | ENST00000438447.1 | PDZ domain containing 2 |
| AGO4 | ENST00000373210.3 | argonaute RISC catalytic component 4 |
| CD97 | ENST00000357355.3 | CD97 molecule |
| EGR3 | ENST00000519492.1 | early growth response 3 |
| PANK3 | ENST00000239231.6 | pantothenate kinase 3 |
| IL6R | ENST00000344086.4 | interleukin 6 receptor |
| DNAJA2 | ENST00000317089.5 | DnaJ (Hsp40) homolog, subfamily A, member 2 |
| GPC4 | ENST00000370828.3 | glypican 4 |
| EFNA2 | ENST00000215368.2 | ephrin-A2 |
| FHDC1 | ENST00000260008.3 | FH2 domain containing 1 |
| SNX29 | ENST00000566228.1 | sorting nexin 29 |
| UBN2 | ENST00000473989.3 | ubinuclein 2 |
| RSRC2 | ENST00000331738.7 | arginine/serine-rich coiled-coil 2 |
| AP5M1 | ENST00000261558.3 | adaptor-related protein complex 5, mu 1 subunit |
| RASA1 | ENST00000456692.2 | RAS p21 protein activator (GTPase activating protein) 1 |
| MYCL | ENST00000397332.2 | v-myc avian myelocytomatosis viral oncogene lung carcinoma derived homolog |
| POM121C | ENST00000257665.5 | POM121 transmembrane nucleoporin C |
| ROBO2 | ENST00000461745.1 | roundabout, axon guidance receptor, homolog 2 (Drosophila) |
| TRPM7 | ENST00000560955.1 | transient receptor potential cation channel, subfamily M, member 7 |
| KCNK10 | ENST00000340700.5 | potassium channel, subfamily K, member 10 |
| RDX | ENST00000343115.4 | radixin |
| TGFBR2 | ENST00000359013.4 | transforming growth factor, beta receptor II (70/80kDa) |
| HGF | ENST00000222390.5 | hepatocyte growth factor (hepapoietin A; scatter factor) |
| MARCH6 | ENST00000274140.5 | membrane-associated ring finger (C3HC4) 6, E3 ubiquitin protein ligase |
| ALX4 | ENST00000329255.3 | ALX homeobox 4 |
| FCHO2 | ENST00000430046.2 | FCH domain only 2 |
| MEIS1 | ENST00000488550.1 | Meis homeobox 1 |
| BMPR2 | ENST00000374574.2 | bone morphogenetic protein receptor, type II (serine/threonine kinase) |
| RHOB | ENST00000272233.4 | ras homolog family member B |
| GNG12 | ENST00000370982.3 | guanine nucleotide binding protein (G protein), gamma 12 |
| C16orf52 | ENST00000542527.2 | chromosome 16 open reading frame 52 |
| KIAA1468 | ENST00000398130.2 | KIAA1468 |
| PRKCE | ENST00000306156.3 | protein kinase C, epsilon |
| MTMR12 | ENST00000280285.5 | myotubularin related protein 12 |
| EXTL3 | ENST00000220562.4 | exostosin-like glycosyltransferase 3 |
| KLF3 | ENST00000261438.5 | Kruppel-like factor 3 (basic) |
| BCL2 | ENST00000398117.1 | B-cell CLL/lymphoma 2 |
| RASGRP1 | ENST00000559830.1 | RAS guanyl releasing protein 1 (calcium and DAG-regulated) |
| SCML2 | ENST00000398048.3 | sex comb on midleg-like 2 (Drosophila) |
| BNC2 | ENST00000380672.4 | basonuclin 2 |
| BAHD1 | ENST00000416165.1 | bromo adjacent homology domain containing 1 |
| LMBR1 | ENST00000353442.5 | limb development membrane protein 1 |
| SOX7 | ENST00000554914.1 | Transcription factor SOX-7; Uncharacterized protein; cDNA FLJ58508, highly similar to Transcription factor SOX-7 |
| SLC30A10 | ENST00000366926.3 | solute carrier family 30, member 10 |
| GAB1 | ENST00000262995.4 | GRB2-associated binding protein 1 |
| MSL1 | ENST00000398532.4 | male-specific lethal 1 homolog (Drosophila) |
| COQ10B | ENST00000263960.2 | coenzyme Q10 homolog B (S. cerevisiae) |
| GXYLT1 | ENST00000398675.3 | glucoside xylosyltransferase 1 |
| CREBRF | ENST00000540014.1 | CREB3 regulatory factor |
| SASH1 | ENST00000367467.3 | SAM and SH3 domain containing 1 |
| XKR4 | ENST00000327381.6 | XK, Kell blood group complex subunit-related family, member 4 |
| DCUN1D3 | ENST00000324344.4 | DCN1, defective in cullin neddylation 1, domain containing 3 |
| STK40 | ENST00000359297.2 | serine/threonine kinase 40 |
| PURB | ENST00000395699.2 | purine-rich element binding protein B |
| PLD1 | ENST00000342215.6 | phospholipase D1, phosphatidylcholine-specific |
| CNOT6 | ENST00000393356.1 | CCR4-NOT transcription complex, subunit 6 |
| MCAM | ENST00000392814.1 | melanoma cell adhesion molecule |
| CREBL2 | ENST00000228865.2 | cAMP responsive element binding protein-like 2 |
| KCNA1 | ENST00000382545.3 | potassium voltage-gated channel, shaker-related subfamily, member 1 (episodic ataxia with myokymia) |
| CDK6 | ENST00000265734.4 | cyclin-dependent kinase 6 |
| SCRN1 | ENST00000242059.5 | secernin 1 |
| NBEA | ENST00000379939.2 | neurobeachin |
| SEMA3A | ENST00000265362.4 | sema domain, immunoglobulin domain (Ig), short basic domain, secreted, (semaphorin) 3A |
| TMEM260 | ENST00000261556.6 | transmembrane protein 260 |
| MEF2C | ENST00000340208.5 | myocyte enhancer factor 2C |
| TGFB2 | ENST00000366930.4 | transforming growth factor, beta 2 |
| TNKS | ENST00000310430.6 | tankyrase, TRF1-interacting ankyrin-related ADP-ribose polymerase |
| RBMS3 | ENST00000396583.3 | RNA binding motif, single stranded interacting protein 3 |
| TNPO1 | ENST00000337273.5 | transportin 1 |
| SUZ12 | ENST00000322652.5 | SUZ12 polycomb repressive complex 2 subunit |
| AP3M1 | ENST00000355264.4 | adaptor-related protein complex 3, mu 1 subunit |
| EHD1 | ENST00000320631.3 | EH-domain containing 1 |
| DNAJB14 | ENST00000442697.2 | DnaJ (Hsp40) homolog, subfamily B, member 14 |
| CD47 | ENST00000361309.5 | CD47 molecule |
| ATXN3 | ENST00000545170.1 | ataxin 3 |
| C17orf102 | ENST00000357754.1 | chromosome 17 open reading frame 102 |
| ABCD2 | ENST00000308666.3 | ATP-binding cassette, sub-family D (ALD), member 2 |
| ACVR2A | ENST00000241416.7 | activin A receptor, type IIA |
| MPRIP | ENST00000341712.4 | myosin phosphatase Rho interacting protein |
| SNX19 | ENST00000265909.4 | sorting nexin 19 |
| PAIP2B | ENST00000244221.8 | poly(A) binding protein interacting protein 2B |
| WNT2B | ENST00000369686.5 | wingless-type MMTV integration site family, member 2B |
| BCL11B | ENST00000357195.3 | B-cell CLL/lymphoma 11B (zinc finger protein) |
| ZNF207 | ENST00000394670.4 | zinc finger protein 207 |
| TRIM33 | ENST00000358465.2 | tripartite motif containing 33 |
| TSPAN2 | ENST00000369516.2 | tetraspanin 2 |
| MAP3K8 | ENST00000375321.1 | mitogen-activated protein kinase kinase kinase 8 |
| ANKRD33B | ENST00000296657.5 | ankyrin repeat domain 33B |
| BMP3 | ENST00000282701.2 | bone morphogenetic protein 3 |
| STRN | ENST00000263918.4 | striatin, calmodulin binding protein |
| ACBD5 | ENST00000396271.3 | acyl-CoA binding domain containing 5 |
| NETO2 | ENST00000562435.1 | neuropilin (NRP) and tolloid (TLL)-like 2 |
| KLF9 | ENST00000377126.2 | Kruppel-like factor 9 |
| DAG1 | ENST00000515359.2 | dystroglycan 1 (dystrophin-associated glycoprotein 1) |
| MEGF9 | ENST00000373930.3 | multiple EGF-like-domains 9 |
| RNF24 | ENST00000336095.6 | ring finger protein 24 |
| FAM126B | ENST00000418596.3 | family with sequence similarity 126, member B |
| UNC80 | ENST00000439458.1 | unc-80 homolog (C. elegans) |
| THBD | ENST00000377103.2 | thrombomodulin |
| CCDC171 | ENST00000380701.3 | coiled-coil domain containing 171 |
| RNF111 | ENST00000348370.4 | ring finger protein 111 |
| BRWD3 | ENST00000373275.4 | bromodomain and WD repeat domain containing 3 |
| MRPL45 | ENST00000312513.5 | mitochondrial ribosomal protein L45 |
| MAPK1 | ENST00000215832.6 | mitogen-activated protein kinase 1 |
| PYGO1 | ENST00000302000.6 | pygopus homolog 1 (Drosophila) |
| TBX2 | ENST00000240328.3 | T-box 2 |
| TNRC6B | ENST00000335727.9 | trinucleotide repeat containing 6B |
| IFFO2 | ENST00000455833.2 | intermediate filament family orphan 2 |
| MTAP | ENST00000380172.4 | methylthioadenosine phosphorylase |
| NIPBL | ENST00000448238.2 | Nipped-B homolog (Drosophila) |
| HECTD1 | ENST00000553700.1 | HECT domain containing E3 ubiquitin protein ligase 1 |
| SNTB2 | ENST00000336278.4 | syntrophin, beta 2 (dystrophin-associated protein A1, 59kDa, basic component 2) |
| C17orf75 | ENST00000577809.1 | chromosome 17 open reading frame 75 |
| ZADH2 | ENST00000322342.3 | zinc binding alcohol dehydrogenase domain containing 2 |
| TIGD2 | ENST00000317005.2 | tigger transposable element derived 2 |
| HDAC9 | ENST00000405010.3 | histone deacetylase 9 |
| HNRNPU | ENST00000444376.2 | heterogeneous nuclear ribonucleoprotein U (scaffold attachment factor A) |
| KLF12 | ENST00000377669.2 | Kruppel-like factor 12 |
| TNS1 | ENST00000171887.4 | tensin 1 |
| SAMD8 | ENST00000372687.4 | sterile alpha motif domain containing 8 |
| RPS6KA3 | ENST00000379565.3 | ribosomal protein S6 kinase, 90kDa, polypeptide 3 |
| FOXP2 | ENST00000408937.3 | forkhead box P2 |
| GID4 | ENST00000268719.4 | GID complex subunit 4 |
| AAK1 | ENST00000409085.4 | AP2 associated kinase 1 |
| DNAJC16 | ENST00000375847.3 | DnaJ (Hsp40) homolog, subfamily C, member 16 |
| TNPO3 | ENST00000393245.1 | transportin 3 |
| SPRY4 | ENST00000344120.4 | sprouty homolog 4 (Drosophila) |
| PRPF4B | ENST00000337659.6 | pre-mRNA processing factor 4B |
| FOXP1 | ENST00000318789.4 | forkhead box P1 |
| WNK3 | ENST00000375169.3 | WNK lysine deficient protein kinase 3 |
| AGO2 | ENST00000220592.5 | argonaute RISC catalytic component 2 |
| SOX6 | ENST00000316399.6 | SRY (sex determining region Y)-box 6 |
| NHS | ENST00000380060.3 | Nance-Horan syndrome (congenital cataracts and dental anomalies) |
| PTPN9 | ENST00000306726.2 | protein tyrosine phosphatase, non-receptor type 9 |
| YAP1 | ENST00000282441.5 | Yes-associated protein 1 |
| ETNK1 | ENST00000266517.4 | ethanolamine kinase 1 |
| SNX11 | ENST00000393405.2 | sorting nexin 11 |
| HSPA13 | ENST00000285667.3 | heat shock protein 70kDa family, member 13 |
| GTPBP10 | ENST00000222511.6 | GTP-binding protein 10 (putative) |
| RAB11A | ENST00000569896.1 | RAB11A, member RAS oncogene family |
| FAT3 | ENST00000298047.6 | FAT atypical cadherin 3 |
| DST | ENST00000312431.6 | dystonin |
| CRIM1 | ENST00000280527.2 | cysteine rich transmembrane BMP regulator 1 (chordin-like) |
| PDE3A | ENST00000359062.3 | phosphodiesterase 3A, cGMP-inhibited |
| FMN1 | ENST00000334528.9 | formin 1 |
| SESN3 | ENST00000536441.1 | sestrin 3 |
| NFAT5 | ENST00000354436.2 | nuclear factor of activated T-cells 5, tonicity-responsive |
| KAT6A | ENST00000265713.2 | K(lysine) acetyltransferase 6A |
| FOXG1 | ENST00000382535.3 | forkhead box G1 |
| SPPL3 | ENST00000353487.2 | signal peptide peptidase like 3 |
| SEMA3E | ENST00000307792.3 | sema domain, immunoglobulin domain (Ig), short basic domain, secreted, (semaphorin) 3E |
| TENM4 | ENST00000278550.7 | teneurin transmembrane protein 4 |
| TRMT5 | ENST00000261249.6 | tRNA methyltransferase 5 |
| FAM107B | ENST00000378470.1 | family with sequence similarity 107, member B |
| MCMBP | ENST00000360003.3 | minichromosome maintenance complex binding protein |
| FTO | ENST00000471389.1 | fat mass and obesity associated |
| CDKN2C | ENST00000262662.1 | cyclin-dependent kinase inhibitor 2C (p18, inhibits CDK4) |
| C1orf112 | ENST00000359326.4 | chromosome 1 open reading frame 112 |
| MTHFD1 | ENST00000216605.8 | methylenetetrahydrofolate dehydrogenase (NADP+ dependent) 1, methenyltetrahydrofolate cyclohydrolase, formyltetrahydrofolate synthetase |
| RETSAT | ENST00000295802.4 | retinol saturase (all-trans-retinol 13,14-reductase) |
| CMC1 | ENST00000466830.1 | COX assembly mitochondrial protein 1 homolog (S. cerevisiae) |
| CYB5R4 | ENST00000369681.5 | cytochrome b5 reductase 4 |
| CADM1 | ENST00000452722.3 | cell adhesion molecule 1 |
| BCAS3 | ENST00000589222.1 | breast carcinoma amplified sequence 3 |
| TLK2 | ENST00000582809.1 | tousled-like kinase 2 |
| FZD8 | ENST00000374694.1 | frizzled family receptor 8 |
| METAP1 | ENST00000296411.6 | methionyl aminopeptidase 1 |

* TargetScan predicts biological targets of miRNAs by searching for the presence of conserved 8mer, 7mer, and 6mer sites that match the seed region of each miRNA
