# Supplementary material for: MicroRNA-21 induces cisplatin resistance in head and neck squamous cell carcinoma
Source: PLoS One. 2022 Apr 14;17(4):e0267017. doi: 10.1371/journal.pone.0267017 (PMC9009694; doi:10.1371/journal.pone.0267017)
Supplement: S1 Raw images — (PDF) [file pone.0267017.s006.pdf]

Fig.1.A Agarose gel electrophoresis

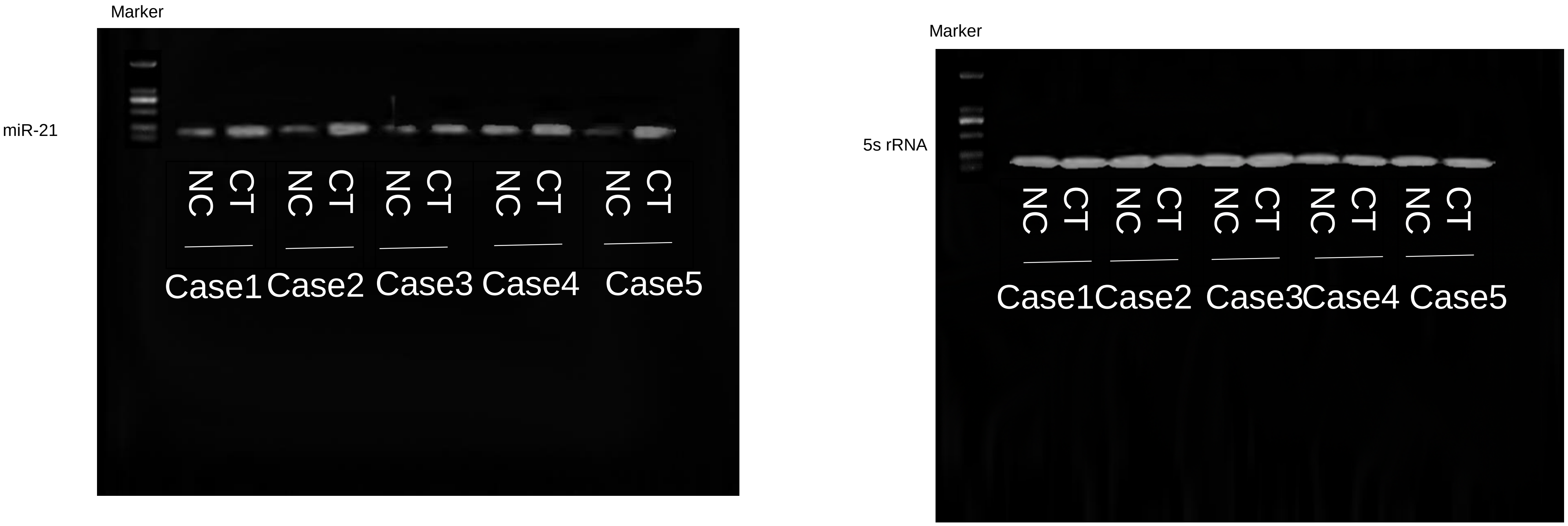

# Fig.1.A Agarose gel electrophoresis

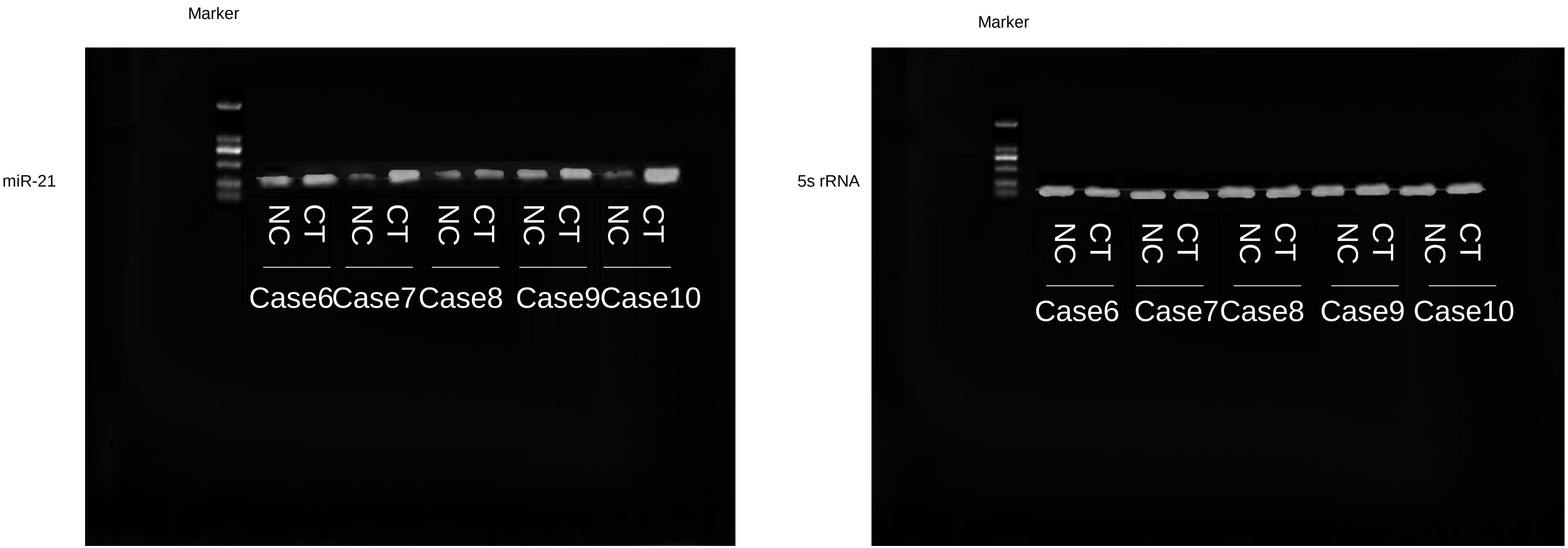

Fig.1.D Agarose gel electrophoresis

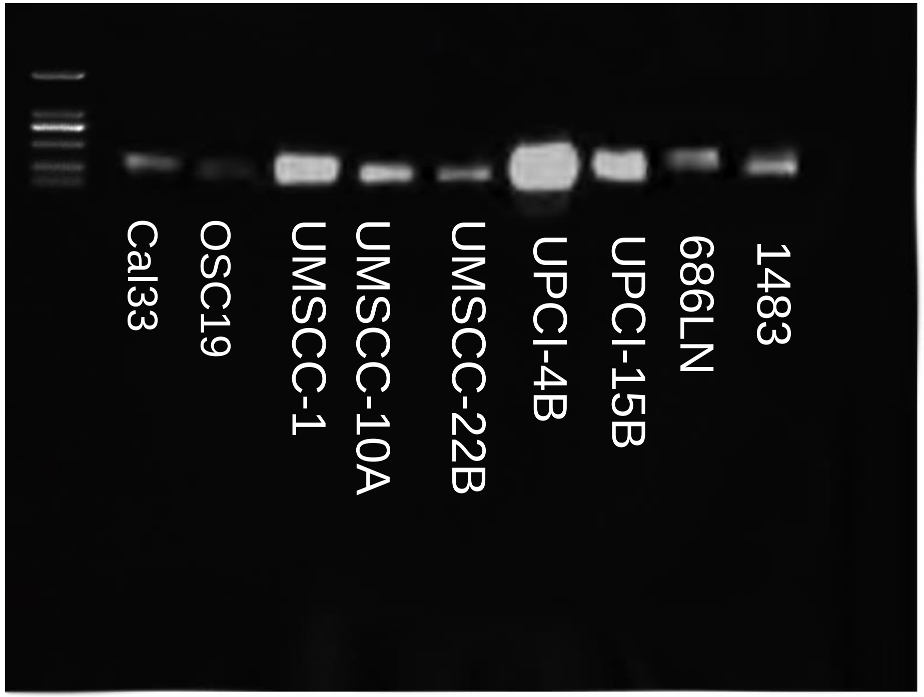


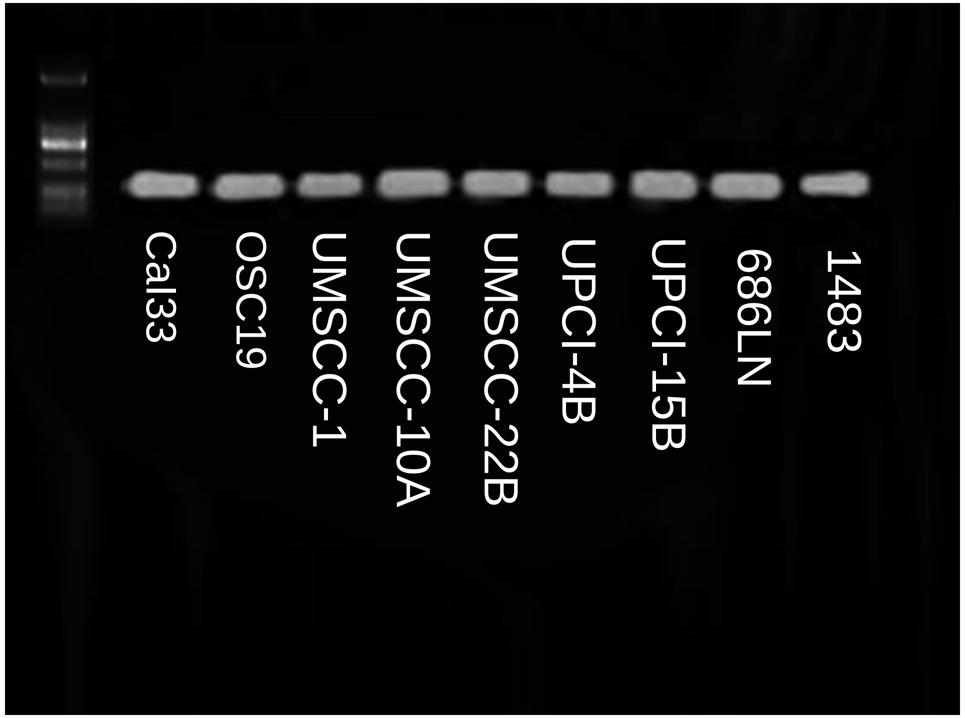

# Fig.2.A Agarose gel electrophoresis UMSCC-1

Marker

Has-miR-21

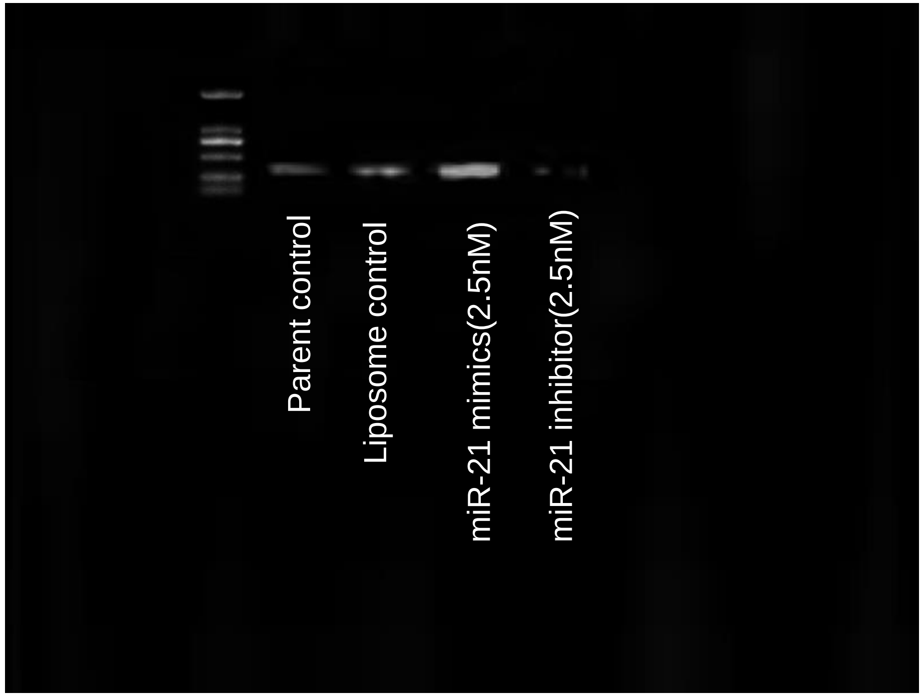


Marker

5s rRNA

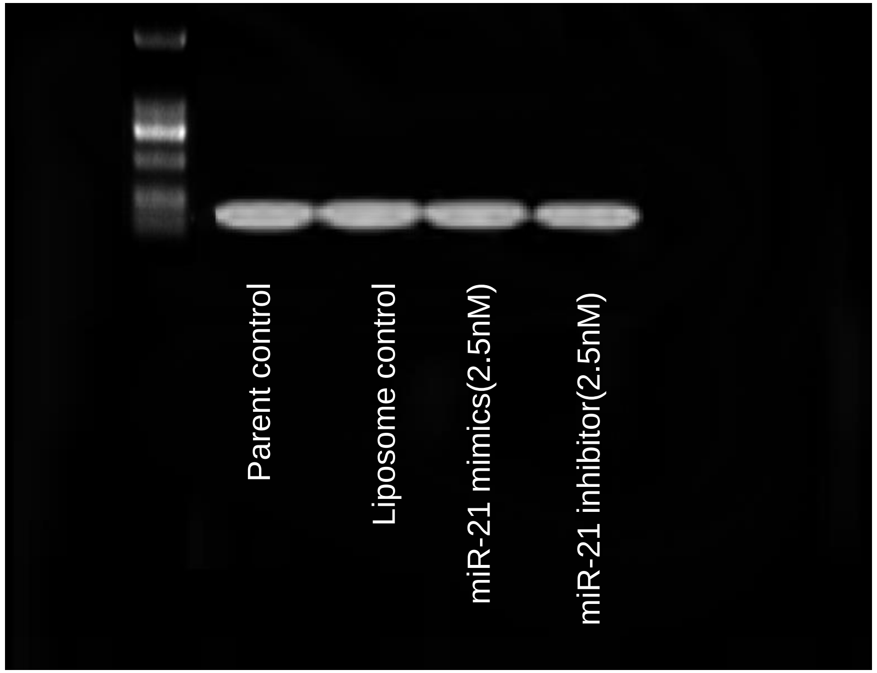

# Agarose gel electrophoresis

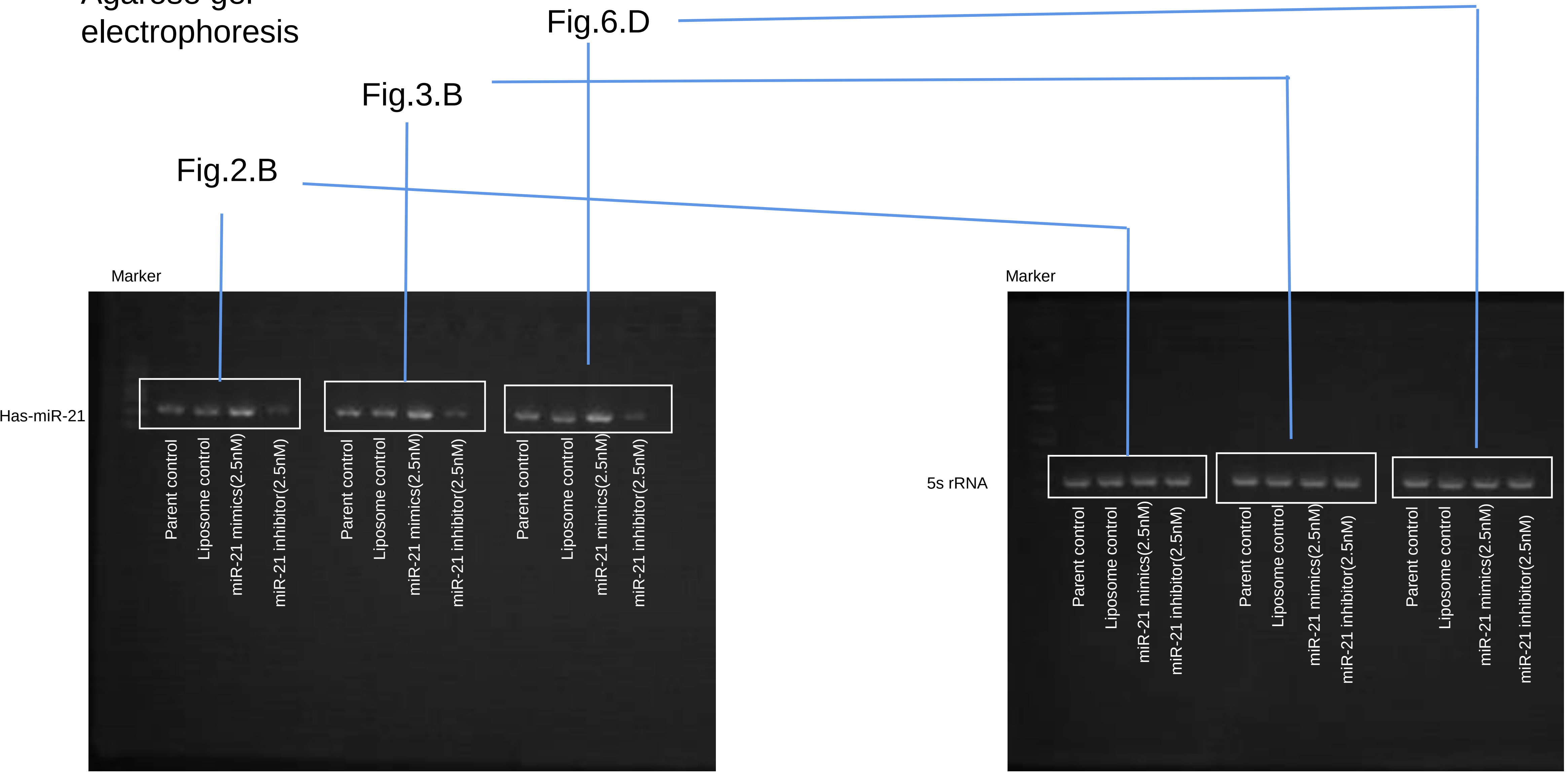

Fig.3.A Agarose gel electrophoresis UMSCC-1

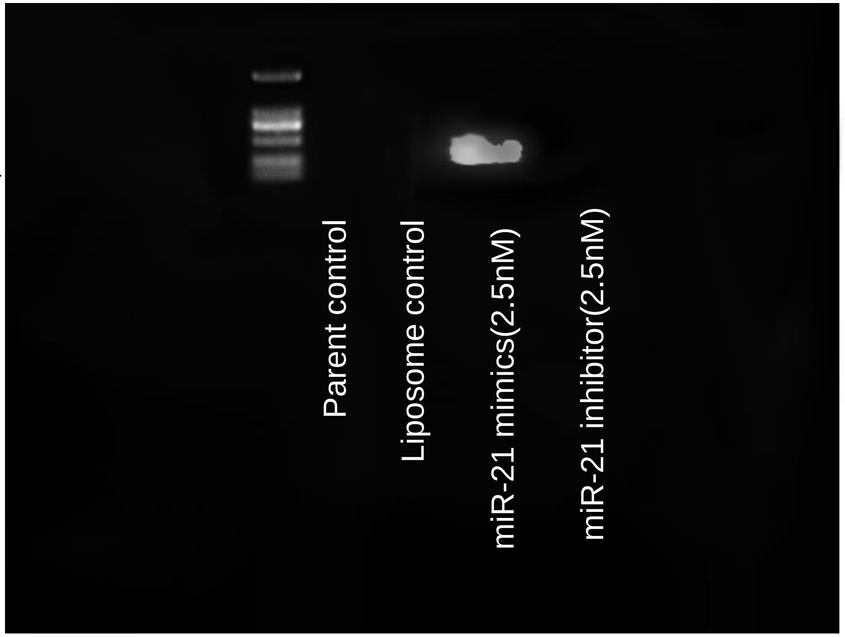


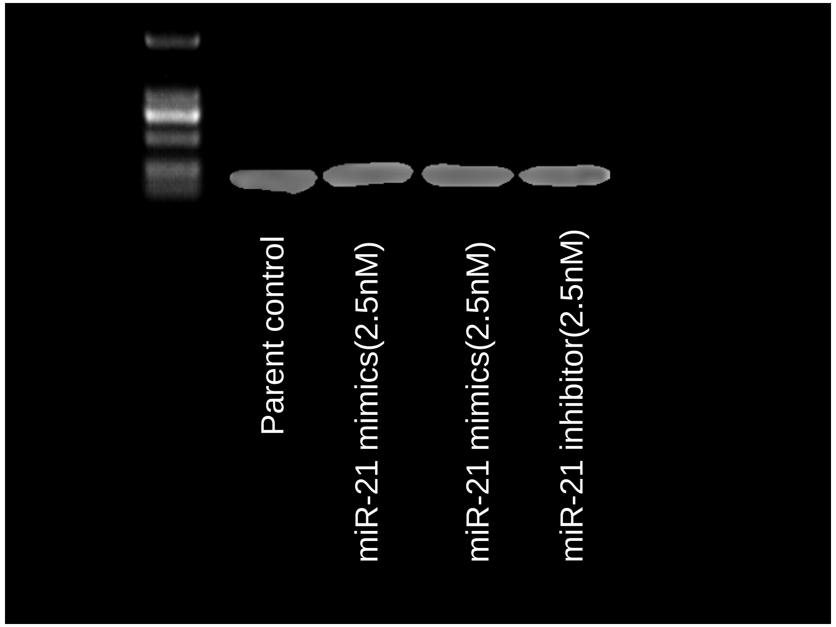

Fig.6.B     Agarose gel electrophoresis     UMSCC-1

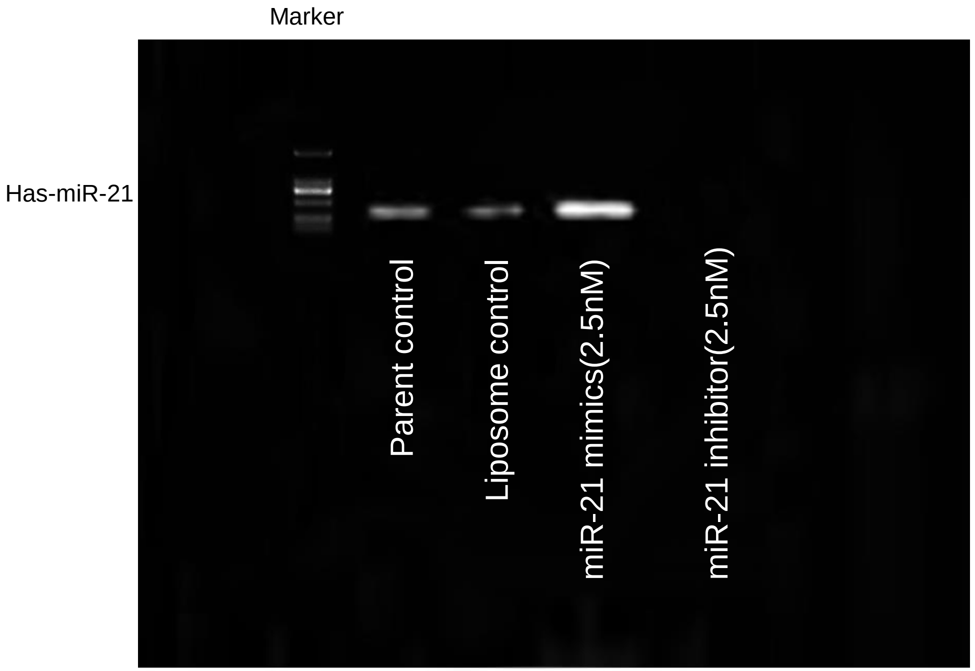


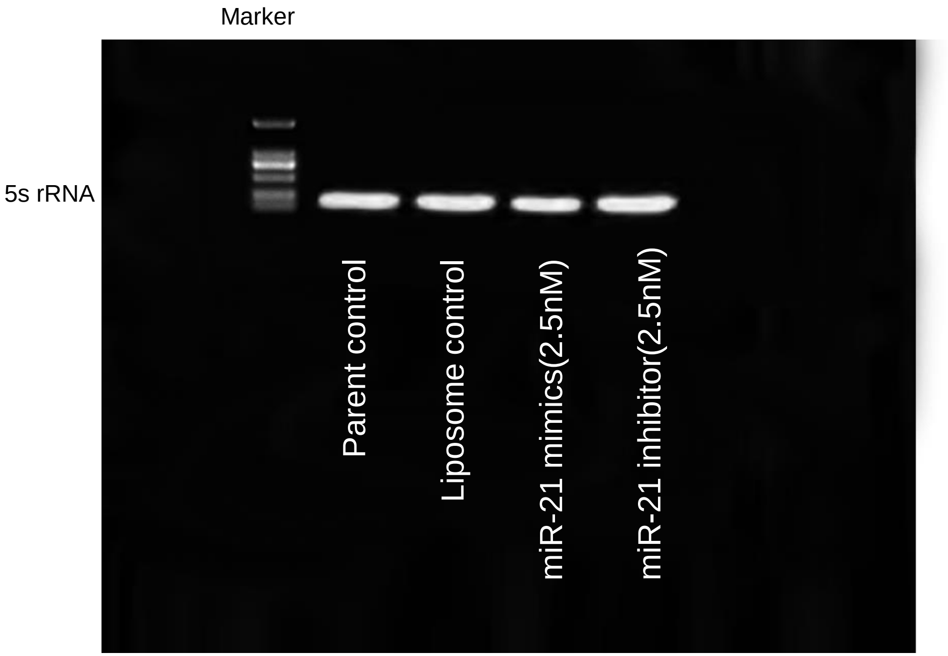

Fig.6.C

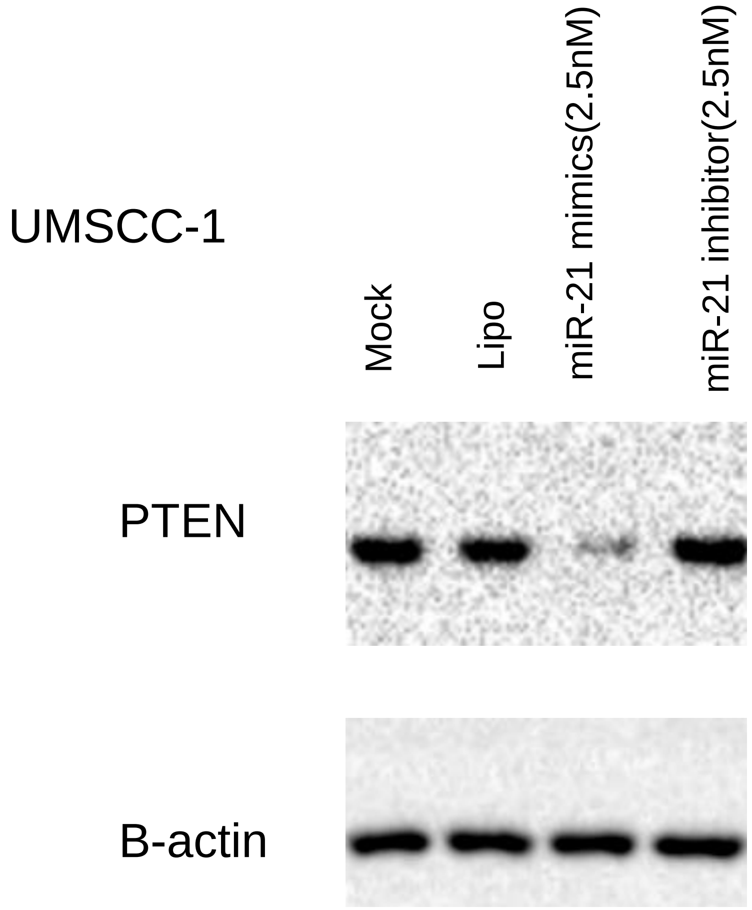


Western Blot

Fig.6.E

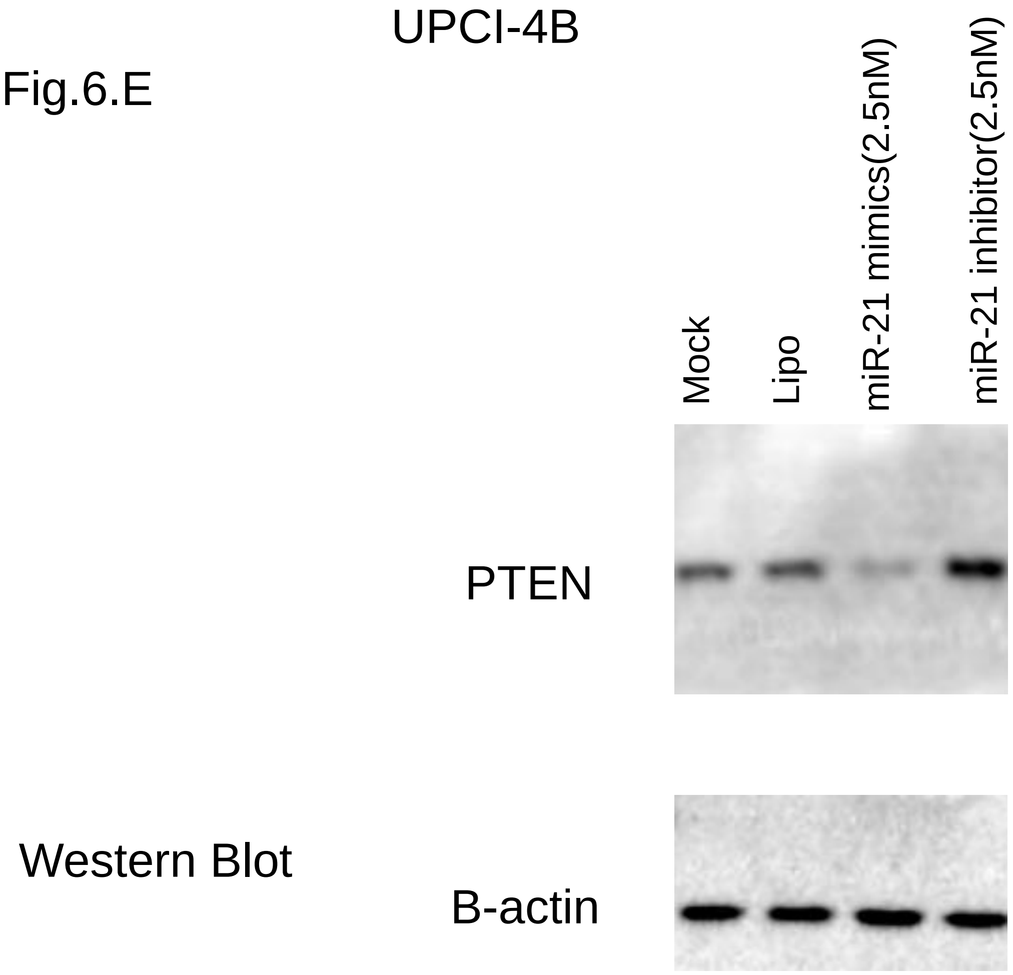
UPCI-4B
Mock
Lipo
miR-21 mimics(2.5nM)
miR-21 inhibitor(2.5nM)
PTEN
Western Blot
B-actin
